# Design of a Magnetic Nanoplatform Based on CD26 Targeting and HSP90 Inhibition for Apoptosis and Ferroptosis-Mediated Elimination of Senescent Cells

**DOI:** 10.1021/acsbiomaterials.4c00771

**Published:** 2024-12-04

**Authors:** Maciej Wnuk, Susel Del Sol-Fernández, Dominika Błoniarz, Julia Słaby, Tomasz Szmatoła, Michał Żebrowski, Pablo Martínez-Vicente, Grzegorz Litwinienko, María Moros, Anna Lewińska

**Affiliations:** a Institute of Biotechnology, College of Natural Sciences, University of Rzeszow, Pigonia 1, Rzeszow 35-310, Poland; b Instituto de Nanociencia y Materiales de Aragón, INMA (CSIC-Universidad de Zaragoza), C/Pedro Cerbuna 12, Zaragoza 50009, Spain; c Doctoral School, University of Rzeszow, Rejtana 16C, Rzeszow 35-959, Poland; d Center of Experimental and Innovative Medicine, University of Agriculture in Krakow, al. Mickiewicza 24/28, Cracow 30-059, Poland; e Faculty of Chemistry, University of Warsaw, Pasteura 1, Warsaw 02-093, Poland; f Centro de Investigación Biomédica en Red de Bioingeniería, Biomateriales y Nanomedicina (CIBER-BBN), Madrid 28029, Spain

**Keywords:** iron oxide nanoparticles, CD26, HSP90 inhibitor, drug-induced senescence, skin cells, senolysis

## Abstract

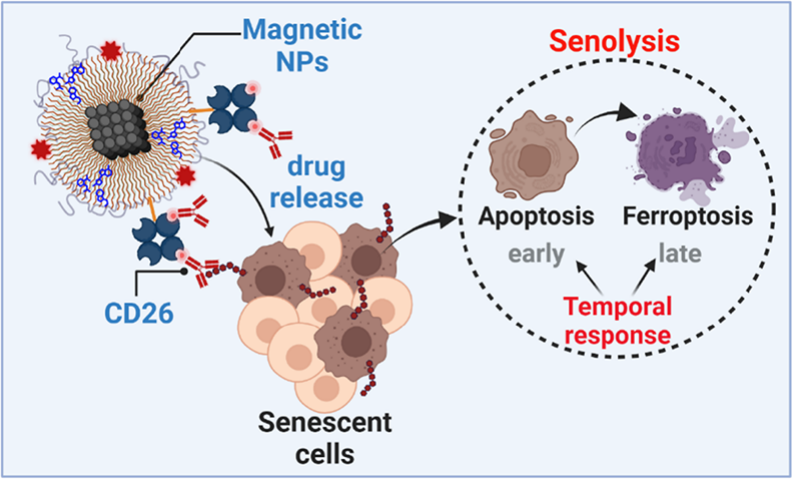

The accumulation of senescent cells, a hallmark of aging and age-related diseases, is also considered as a side effect of anticancer therapies, promoting drug resistance and leading to treatment failure. The use of senolytics, selective inducers of cell death in senescent cells, is a promising pharmacological antiaging and anticancer approach. However, more studies are needed to overcome the limitations of first-generation senolytics by the design of targeted senolytics and nanosenolytics and the validation of their usefulness in biological systems. In the present study, we have designed a nanoplatform composed of iron oxide nanoparticles functionalized with an antibody against a cell surface marker of senescent cells (CD26), and loaded with the senolytic drug HSP90 inhibitor 17-DMAG (MNP@CD26@17D). We have documented its action against oxidative stress-induced senescent human fibroblasts, WI-38 and BJ cells, and anticancer drug-induced senescent cutaneous squamous cell carcinoma A431 cells, demonstrating for the first time that CD26 is a valid marker of senescence in cancer cells. A dual response to MNP@CD26@17D stimulation in senescent cells was revealed, namely, apoptosis-based early response (2 h treatment) and ferroptosis-based late response (24 h treatment). MNP@CD26@17D-mediated ferroptosis might be executed by ferritinophagy as judged by elevated levels of the ferritinophagy marker NCOA4 and a decreased pool of ferritin. As 24 h treatment with MNP@CD26@17D did not induce hemolysis in human erythrocytes *in vitro*, this newly designed nanoplatform could be considered as an optimal multifunctional tool to target and eliminate senescent cells of skin origin, overcoming their apoptosis resistance.

## 1 Introduction

Cellular senescence, a stress response characterized by permanent cell cycle arrest and a plethora of morphological, biochemical, and molecular traits, may promote both beneficial and detrimental effects, depending on the biological context.^1,2^ Indeed, although initially considered as a tumor suppression mechanism, when senescent cells with elevated secretion of proinflammatory factors (senescence-associated secretory phenotype, SASP) are accumulated in aged tissues, they could be implicated in inflammation-driven secondary senescence, tumor promotion, and progression.^3,4^ Although the activation of a senescence program is normally a result of replicative exhaustion in normal cells (telomere loss-mediated replicative senescence), different physical and chemical stress stimuli may also trigger stress-induced senescence in both normal and cancer cells.^5^ Indeed, cellular senescence is also considered as a side effect of chemotherapy, ultimately limiting the elimination of both nonsenescent and senescent cancer cells.^4,6^

To date, two experimental approaches have been successfully developed to clear senescent cells *in vitro* and *in vivo*, by means of genetic methods and pharmacological agents, respectively.^7,8^ Several classes of senolytic drugs with the potential to interfere with cell survival pathways and selectively kill senescent cells have been documented in preclinical *in vitro* and mouse models.^9,10^ Among them, naturally occurring polyphenols (e.g., quercetin), Bcl-2 protein inhibitors, kinase inhibitors, and other repurposed anticancer drugs (e.g., dasatinib), p53 stabilizers, heat shock protein (HSP) inhibitors, and cardiac glycosides along with their healthspan promoting effects have been described.

However, systemic administration of the first-generation senolytics may promote some adverse off-target effects such as thrombocytopenia and neutropenia.^6^ Therefore, more studies on senolytic design and testing are needed to improve the specific targeting of senescent cells in order to decrease the side effects.

Among different approaches to improve the efficiency and safety of senolytics, the use of antibody-drug conjugates has been reported.^11^ For example, an antibody that recognizes senescence-associated membrane marker B2M and conjugated to duocarmycin, a chemotherapeutic drug, selectively killed senescent cancer cells with no toxicity to proliferating cells.^11^ Similarly, the use of nanocarriers functionalized with specific moieties to selectively recognize senescent cells can also be considered as a promising strategy. The possibility to increase the bioavailability and safety profiles of senolytics and the opportunity to design senolytic-based theranostic nanocarriers make this approach appealing for translational medicine.^10,12^

In recent years, there have been several attempts to synthesize and validate the usefulness of multifunctional nanobased tools such as mesoporous silica nanoparticles, carbon quantum dots, calcium carbonate nanoparticles, and molecularly imprinted polymer nanoparticles in senotherapies.^13−15^ For example, to identify senescent cells and promote antisenescence effects, CD9 monoclonal antibody-conjugated lactose-wrapped calcium carbonate nanoparticles loaded with rapamycin (CD9-Lac/CaCO_3_/Rapa) were designed and tested.^16^ The anti-CD9 antibody detected senescent cells with overexpressed CD9 on the cell surface, while CaCO_3_ nanoparticles delivered rapamycin to exert anti-inflammatory action in senescent cells by lowering the levels of SASP components (so-called senostatic/senomorphic activity).^16^

In view of these promising results and to further expand the use of antibody-based nanosenolytics and nanosenostatics, a comprehensive functional analysis of the surfaceome of senescent cells is still needed.^17^

Among the surface biomarkers already reported to be present in normal and senescent cells is dipeptidyl peptidase 4 (DPP4/cluster of differentiation 26, CD26), a transmembrane glycoprotein. CD26 is expressed on numerous cell types and is considered as a moonlighting protein with multiple biological functions based on its enzymatic peptidase activity against growth factors, chemokines, neuropeptides, and hormones, and the possibility of protein–protein interactions.^18−20^ Indeed, CD26 has been implicated in the regulation of immune responses, glucose metabolism, signal transduction, cell death pathways, and cancer progression.^18−20^ CD26 has also been proposed as a senescence-associated cell surface marker in normal human fibroblasts.^21^ However, it is unknown whether CD26 levels are also elevated in drug-induced senescent cancer cells and if CD26 can be targeted during chemotherapy-associated senescence.

Magnetic iron-based nanoparticles (MNPs) have been used in numerous biomedical applications due to their intrinsic magnetic properties, along with their size and large surface area, which allows the possibility of functionalization. For instance, MNPs can be used as contrast agents for magnetic resonance imaging and as death-promoting agents to kill cancer cells by heating upon the application of an alternating magnetic field, which is known as magnetic hyperthermia.^22^ MNPs can be also helpful for the elimination of apoptosis-insensitive drug-resistant cancer cells by means of iron-induced lipid peroxidation-mediated regulated cell death, namely, ferroptosis.^22,23^

In the present study, we have designed and synthesized a multifunctional nanoplatform based on iron oxide nanoparticles conjugated with an anti-CD26 antibody and loaded with 17-DMAG, an HSP90 inhibitor, to specifically target and eliminate human senescent cells. We have tested the nanoplatform in both normal (lung and skin fibroblasts) and tumoral (skin cancer) senescent cells. We have documented that CD26 levels can also be increased as a result of chemotherapy-induced senescence in skin cancer cells and that the nanoplatform can sensitize drug-induced senescent cancer cells to cell death. The nanoplatform-mediated cell death modality and related mechanisms are also presented and discussed.

## 2 Materials and Methods

### 2.1 Materials

All reagents were commercially purchased and used as received unless otherwise indicated. Iron(III) acetylacetonate (Fe(acac)_3_), oleic acid (OA), PMAO (MW: 30,000–50,000 g/mol), 1,2-dihydroxybenzene-3,5-disulfonic acid (Tiron), sulfo-*N*-hydroxysuccinimide (S-NHS), Amicon ultracentrifugal filters and mixed cellulose esters (MCE) membrane (GSWP04700) were purchased from Merck KGaA (Darmstadt, Germany). α-Methoxy-ω-amino poly(ethylene glycol) (PEG, MW: 750 Da) was purchased from Rapp Polymere GmbH (Tübingen, Germany). PD-10 desalting columns packed with Sephadex G-25 resin were obtained from Cytiva (Barcelona, Spain). 5(6)-TAMRA cadaverine [tetramethylrhodamine 5-(and-6)-carboxamide cadaverine] was obtained from AnaSpec (Fremont, CA, USA). Benzyl ether, hexane, chloroform stabilized with ethanol, 1-ethyl-3-(3-dimethyl aminopropyl)carbodiimide (EDC), Bradford reagent assay, bovine serum albumin standard (BSA), antihuman CD26 biotin monoclonal antibody produced in mouse (BMS143BT), iron standard solution (1 mg/mL in 2 to 5% HNO_3_), and SuperSignal West Femto Maximum Sensitivity Substrate were obtained from Thermo Fisher Scientific (Waltham, MA, USA). Polyclonal goat antimouse immunoglobulin/HRP (secondary antibody) was purchased from Dako (P0447, Glostrup, Denmark). 17-AAG (17-(allylamino)-17-demethoxygeldanamycin, A220462) was purchased from AmBeed (Arlington Hts, IL, USA) and 17-DMAG (17-desmethoxy-17-*N*,*N*-dimethylaminoethylamino-geldanamycin, AA43412) was obtained from Biosynth (Staad, Switzerland).

### 2.2 Synthesis, Functionalization, and Bioconjugation of Magnetic Nanoparticles (MNPs)

#### 2.2.1 Synthesis of Hydrophobic Fe_3_O_4_ MNPs (MNP@OA)

Iron oxide (Fe_3_O_4_) MNPs were synthesized by a one-step thermal decomposition method.^24^ The synthesis was carried out by using a standard Schlenk line. In brief, Fe(acac)_3_ (15 mmol), oleic acid (OA) (45 mmol), 1,2-hexadecanediol (30 mmol), and 150 mL of benzyl ether were mixed and mechanically stirred (100 rpm) under a nitrogen flow. The mixture was heated to 200 °C at a heating rate of 3 °C/min for 1 h, then to 285 °C at a heating rate of 5 °C/min for 2 h. The resulting solution was cooled to room temperature. Under ambient conditions, an excess of ethanol was added to the mixture. A black material was precipitated and magnetically separated using a NdFeB magnet. The black product was dissolved in hexane, precipitated with ethanol, and magnetically separated again. This cycle was repeated three times, and the final solution was dispersed in hexane and kept at 4 °C.

#### 2.2.2 Transference to Water by Polymer Addition Strategy (MNP@PMAO)

The transference into water was performed following a previously reported method with slight modifications.^25^ In brief, 225 mg of poly(maleic anhydride-*alt*-1-octadecene) (PMAO) was added to a flask containing 195 mL of chloroform and placed in an ultrasonic bath for 30 min at room temperature. Subsequently, 10 mg of Fe/mL in 5 mL of CHCl_3_ was added dropwise, and the mixture was sonicated for another 15 min. Afterward, the solvent was slowly removed under a vacuum (200 mbar, 40 °C). MNPs were then resuspended in 20 mL of 0.05 M NaOH and rota-evaporated (200 mbar, 70 °C) to obtain complete evaporation of CHCl_3_. At this point, the solution became clear, as MNPs were completely transferred into water. MNPs were then filtered by using syringe filters of 0.22 μm. To remove the excess of unbound polymer, the MNPs solution was centrifuged at 24,000 rpm for 2 h four times and redispersed in milli-Q water for further use. To allow *in vitro* tracking in a cellular system, MNPs were labeled with a fluorescent dye 5(6)-TAMRA cadaverine [tetramethylrhodamine 5-(and-6)-carboxamide cadaverine].^26^ To do so, 1% of the polymer monomers were modified with TAMRA (2 mg/mL) under magnetic stirring overnight in chloroform before adding the MNPs.

#### 2.2.3 Functionalization with streptavidin and passivation of the surface with PEG (MNP@STV)

To functionalize the MNPs with streptavidin (STV), MNPs (0.5 mg of Fe) were activated with 20 mM EDC and 40 mM S-NHS in MES buffer (100 mM, pH = 6.5) at 37 °C and 60 rpm for 30 min. Then, the excess EDC/S-NHS was removed using a PD-10 desalting column containing Sephadex G-25 resin using gravity flow in phosphate buffer (10 mM, pH = 8). Subsequently, 50 μg/mL of STV was added to the eluted MNPs, and the mixture was incubated at 37 °C for 90 min and stirred at 60 rpm. Afterward, the excess STV was removed using ultracentrifugal filters (Amicon, 100 kDa cutoff). The supernatant was kept for further analysis such as protein determination by the Bradford assay. After three consecutive washing cycles, the particles were resuspended in 800 μL of phosphate buffer and incubated with 200 μL of 10% (w/v) PEG (MW: 750 Da) to block the remaining activated groups. The mixture was kept at 37 °C for 2 h or incubated overnight at 4 °C. At that point, the MNPs were washed from the excess reagents using ultracentrifugal filters (Amicon, 100 kDa cutoff) and stored at 4 °C.

#### 2.2.4 Bioconjugation with Biotin-Modified Monoclonal Antibody (MNP@CD26)

MNPs with STV and passivated with PEG 750 Da (0.3 mg of Fe) were bioconjugated with 2.5 or 10 μg of CD26 monoclonal antibody modified with biotin in 1 mL of PBS pH 7.4 at 37 °C for 90 min. The nonconjugated antibody was removed by centrifugation at 4 °C at 14,500 rpm for 1 h. The supernatant was kept for further analysis.

#### 2.2.5 Drug Loading (MNP@CD26@17A or MNP@CD26@17D)

Different amounts of 17-AAG or 17-DMAG (60, 30, 20, 15, and 12 nmol) were added to the MNP@CD26 (0.1 mg of Fe) and incubated in a total volume of 300 μL of PBS pH 7.4 at room temperature for 3 h. Thereafter, MNPs were centrifuged at 4 °C at 14,500 rpm for 1 h, and the supernatants were collected to determine the amount of drug not incorporated in the MNPs. A calibration curve was performed with 17-AAG or 17-DMAG (serial dilutions from 0 to 125 μg/mL), and the absorbance was recorded at 333 and 331 nm (maximum absorption peaks, respectively) in a UV–VIS–NIR spectrophotometer (V-670, JASCO, Madrid, Spain).

### 2.3 Characterization

#### 2.3.1 MNP Characterization

The MNP size, shape, and distribution were evaluated by TEM using a Tecnai T20 (FEI, Amsterdam, The Netherlands) transmission electron microscope operating at 200 kV. TEM samples were prepared by depositing 5 μL of dilute solution on a copper grid (200 mesh) and posterior drying at ambient temperature before analysis. MNPs size distributions were obtained by measuring more than 200 MNPs by using Fiji software. High-resolution transmission electron microscopy (HRTEM) and scanning transmission electron microscopy (STEM) images were obtained by a Tecnai F30 microscope with an accelerating voltage of 300 kV. The surface chemistry was elucidated from FT-IR spectra using an FT-IR spectrum two spectrometer (PerkinElmer, Madrid, Spain) recorded in the range of 400–4000 cm^–1^. Samples were lyophilized for 24 h before use. Organic contents were determined by thermogravimetric analysis (TGA) using a Universal V4.5A TA instrument (New Castle, DE, USA) under N_2_ atmosphere at a flow rate of 50 mL/min at a rate of 10 °C/min until a final temperature of 800 °C. Hydrodynamic diameters and surface charge were studied by dynamic light scattering (DLS) and ζ-potential measurements using a Malvern Zetasizer Nano instrument. Samples were prepared at a concentration of 0.02 mg of Fe/mL in Milli-Q water and sonicated 10 s before measurement. Each sample was measured five times at 25 °C, combining 10 runs per measurement. For magnetic characterization, the magnetic suspensions were lyophilized and measured as powder, put into a gelatin capsule, and immobilized with cotton wool. Hysteresis loops were measured using a superconducting quantum interference device (SQUID, Quantum Design GmbH, Pfungstadt, Germany) magnetometer at 5 and 300 K fields up to 4000 kA/m.

#### 2.3.2 Determination of Iron Concentration

After each coating, functionalization, and bioconjugation step, the determination of iron concentration was performed following a previously reported protocol.^27^ In brief, 5 μL of MNPs was diluted in 45 μL of solvent (hexane or water) and digested with 100 μL of aqua regia solution (HCl:HNO_3_; 3:1) at 60 °C for 15 min. Then, the samples were diluted up to 300 μL with miliQ water. At this point, 50 μL (in triplicate) was used for the iron quantification by mixing the digested samples with 60 μL of 0.25 M 1,2-dihydroxybenzene-3,5-disulfonic acid (Tiron). This molecule forms a colored complex with iron,^28^ and it can be measured at λ = 480 nm using a microplate spectrophotometer (BioTek Synergy H1 UV/vis, Agilent Technologies, Santa Clara, CA, USA) and compared with a standard calibration curve obtained with solutions of known iron concentrations (0–1000 μg of Fe/mL).

#### 2.3.3 Iron Release from MNPs

The MNPs were dispersed in an artificial lysosomal fluid (Chemazone, BZ257) at an iron concentration of 60 μg/mL. This solution was incubated in a thermomixer (600 rpm, Thermomixer Comfort, Eppendorf) at 37 °C for different time points (2, 6, and 24 h). Then, samples were collected using an Amicon Ultra-0.5 centrifugal filter (Merck KGaA), and the supernatants were digested in HNO_3_ at 60 °C for 1 h. The amount of iron released at each time point was determined by an inductively coupled plasma-optical emission spectrometry (ICP-OES) technique.

#### 2.3.4 Bradford Assay

After functionalizing the MNPs with STV, the supernatants were collected for quantification by the Bradford assay. Briefly, 5 μL of the sample was mixed with 195 μL of Bradford reagent and incubated at room temperature for 10 min before quantification at λ = 595 nm (BioTek Synergy H1 UV/vis microplate spectrophotometer, Agilent Technologies, Santa Clara, CA, USA). Each supernatant was quantified in quadruplicate and compared with a standard curve made with serial dilution of the BSA protein.

#### 2.3.5 Dot Blot

To quantify the amount of antibody bound to the MNPs, 3 μL of the supernatants obtained after the antibody bioconjugation was added to an MCE membrane (0.22 μm) and was left to dry at 37 °C for 30 min. The amount of CD26 antibody (2.5 and 10 μg/mL) used to functionalize the MNPs was considered as a control (100%). At the same time, the MNPs without an antibody were used as additional controls. The membrane was blocked with 2.5% (w/v) bovine serum albumin (BSA) in TBST buffer (Tris-buffered saline (TBS) 1× with 0.1% Tween 20) at 37 °C for 1 h. After blocking, four washing steps were performed using TBST at 37 °C for 5 min. Then, membranes were incubated with a secondary antimouse antibody conjugated with HRP (5 μg/mL) in TBST with 1% (w/v) BSA at room temperature for 2 h. Afterward, 4 washes with TBST (5 min each) and one wash with TBS 1× were performed. Samples were revealed using SuperSignal West Femto kit following the manufacturer’s protocol and the results were visualized using a Chemidoc imaging system (Bio-Rad, Madrid, Spain).

#### 2.3.6 Autoxidation Measurements

The antioxidant behavior of MNPs was evaluated by monitoring the rate of peroxidation in a heterogeneous (micellar) model system *in vitro*. The uptake of dissolved oxygen during peroxidation was carried out at 37 °C at pH 7.4 by using a biological oxygen monitor (Yellow Springs Instruments, Yellow Springs, OH, USA) equipped with a Clark-type electrode. Peroxidation of a sample of 5 mL of aqueous dispersion of methyl linoleate in TritonX-100 micelles was initiated by injection of 100 mL of aqueous solution of 2,2′-azobis(2-methylpropionamidine) dihydrochloride (ABAP, final concentration 10 mM). A detailed description of this experimental system is provided elsewhere.^29,30^

### 2.4 Cell Lines and Culture Conditions

The following human cell lines were used, namely, foreskin fibroblasts (BJ cells, CRL-2522, ATCC, Manassas, VA, USA), fetal lung fibroblasts (WI-38 cells, CCL-75, ATCC, Manassas, VA, USA), and squamous carcinoma A431 cells (85090402, ECACC, Public Health England, Porton Down, Salisbury, UK). Normal and cancer cells were cultured in Dulbecco’s Modified Eagle’s medium (DMEM medium supplemented with 10% FBS, 100 U/mL penicillin, 0.1 mg/mL streptomycin, and 0.25 μg/mL amphotericin B, Corning, Tewksbury, MA, USA) in a cell culture incubator (in a humidified atmosphere containing 5% CO_2_ at 37 °C). Cells were passaged using 0.25% trypsin/2.21 mM EDTA solution (Corning, Tewksbury, MA, USA). Only proliferatively active fibroblasts were used (WI-38 and BJ cells at population doubling levels ≈ 35).^31^ Before the analysis of the senolytic activity of MNP@CD26@17A and MNP@CD26@17D against senescent cells, the effects of free drugs 17-AAG and 17-DMAG, MNP@CD26, MNP@CD26@17A, and MNP@CD26@17D were initially screened in nonsenescent cells using MTT test. Briefly, nonsenescent cells were treated with the drugs (0.1 to 10 μM) or the nanoplatforms (60 μg of Fe/mL) for 24 h (96-well plate, 10,000 cells per a well) and changes in the metabolic activity were investigated using the MTT assay.^32^ Metabolic activity under control conditions (CTR) was considered as 100%. The effects of solvents (methanol for 17-AAG and distilled water for 17-DMAG) were also tested.

### 2.5 Cellular Models of *In Vitro* Senescence

Two models of stress-induced senescence were considered, namely oxidative stress-induced senescence in normal fibroblasts^31^ and anticancer drug-induced senescence in skin cancer cells.^33^ Briefly, to induce premature senescence in normal cells, fibroblasts were stimulated with 100 μM hydrogen peroxide (Merck KGaA, Darmstadt, Germany) for 2 h and cultured without an oxidant for 7 days to develop the senescent phenotype. On the other hand, skin cancer cells were incubated with 50 nM doxorubicin or 1 μM etoposide (Merck KGaA, Darmstadt, Germany) for 24 h to mimic the activation of the senescence program driven by chemotherapy. Similar to normal cells, cancer cells were cultured for up to 7 days to allow the manifestation of a senescent phenotype. Senescent cells were treated with free drugs 17-AAG (100 nM) and 17-DMAG (100 nM), MNPs conjugated with anti-CD26 antibody (MNP@CD26), and MNPs conjugated with anti-CD26 antibody and loaded with 17-AAG (MNP@CD26@17A) or 17-DMAG (MNP@CD26@17D) at concentrations of 30 and/or 60 μg of Fe/mL for 2, 6, and 24 h, and selected parameters were then assayed.

### 2.6 Uptake of the Nanoplatform, Cellular Localization, and Morphological Analysis

To analyze the uptake of the nanoplatforms, namely, MNP@CD26 and MNP@CD26@17D, TAMRA dye was used: MNP@T@CD26 and MNP@T@CD26@17D, respectively. The uptake and cellular localization of nanoplatforms were assessed upon 24 h of incubation of senescent normal and skin cancer cells using imaging flow cytometry (Amnis FlowSight imaging flow cytometer) and IDEAS software (version 6.2.187.0, Luminex Corporation, Austin, TX, USA). Two parameters were considered, namely, maximum pixel and intensity (Ch03). Lysosomal localization of the nanoplatform was confirmed by colocalization analysis of lysosomal senescence-associated beta-galactosidase activity using a CellEvent senescence green detection kit (Thermo Fisher Scientific, Waltham, MA, USA). Moreover, cell morphology was characterized using bright field (BF, Ch01) and two parameters, aspect ratio intensity and area (Ch01). Several subpopulations were revealed based on cell size, cell fragmentation, and damaged cell membrane. Lysosome status was also analyzed using a lysosome marker, namely, GFP-based imaging of Lamp1 (CellLight Lysosomes-GFP, BacMam 2.0 (C10596, Thermo Fisher Scientific, Waltham, MA, USA) as previously described.^34^ GFP-Lamp1 signals were captured using the confocal imaging system IN Cell Analyzer 6500 HS and IN Carta software (Cytiva, Marlborough, MA, USA). GFP-Lamp1 signals (protein levels) are presented in relative fluorescence units (RFU).

### 2.7 Trypan Blue Staining

The necrotic morphotype of cell death in treated cells was documented by using a dye exclusion assay. At selected time points, cells were stained using 0.4% trypan blue solution, and the levels of live (nonstained cells, %) and necrotic (permanently stained cells, %) were calculated using TC10 Automated Cell Counter (Bio-Rad, Hercules, CA, USA).

### 2.8 Annexin V Staining

The apoptotic morphotype of cell death in treated cells was assayed using Annexin V staining as a marker of apoptosis (phosphatidylserine externalization). At selected time points, cells were dual-stained using Annexin V staining (apoptosis) and 7-AAD staining (necrosis) using Muse Annexin V and Dead Cell Assay Kit according to the manufacturer’s instructions (Luminex Corporation, Austin, TX, USA). Muse Cell Analyzer was used to reveal four cell subpopulations [%], namely, live cells (negative for both stainings), early apoptotic cells (Annexin V-positive cells), late apoptotic cells (positive for both stainings), and necrotic cells (7-AAD-positive cells).

### 2.9 Imaging Flow Cytometry and Imaging Cytometry

At selected time points, cells were fixed and immunostained as previously described.^34^ The following primary and secondary antibodies were used, namely anti-CD26 (1:200, MA5–32643), anti-p21 (1:800, MA5–14949), anti-p27 (1:500, PA5–27188), anticaspase 3 (1:500, PA5–77887), anti-HSP90 (1:200, PA3–013), anti-FOXO3a (1:200, MA5–14932), anti-SOD1 (1:200, PA1–30195), anti-GPX4 (1:100, PA5–120674), anti-NRF2 (1:200, PA5–27882), anti-ACSL4 (1:250, PA5–100033), antitransferrin receptor (TfR) (1:250, 13–6890), anti-NCOA4 (1:100, PA5–115626), antiferritin (1:250, MiF2502), anti-NF-κB p65 (1:100, PA5–16545), antirabbit IgG conjugated to Texas Red (TR) (1:1000, T2767), antimouse IgG conjugated to FITC (1:1000, F2761), antirabbit IgG conjugated to FITC (1:1000, F2765), antirabbit IgG conjugated to PE-cyanine 5.5 (1:1000, L42018), and goat antimouse IgG conjugated to cyanine 5 (1:1000, A10524) (Thermo Fisher Scientific, Waltham, MA, USA). For quantitative analysis of protein levels, two imaging approaches were used, namely, imaging flow cytometry (Amnis FlowSight imaging flow cytometer) and IDEAS software (version 6.2.187.0, Luminex Corporation, Austin, TX, USA), and confocal imaging system IN cell analyzer 6500 HS and IN Carta software (Cytiva, Marlborough, MA, USA). Protein levels (total or nuclear) are presented in relative fluorescence units (RFU).

Imaging flow cytometry and CellEvent senescence green detection kit (Thermo Fisher Scientific, Waltham, MA, USA) were used to analyze senescence-associated β-galactosidase (SA-β-gal) activity.^33^ SA-β-gal activity is presented in relative fluorescence units (RFU).

Imaging cytometry and Click-iT Lipid Peroxidation Imaging Kit–Alexa Fluor 488 (C10446, Thermo Fisher Scientific, Waltham, MA, USA) were used to investigate lipid peroxidation-derived protein modifications as previously described.^34^ Lipid peroxidation is presented in relative fluorescence units (RFU).

### 2.10 Glutathione Redox Potential (GSH/GSSG)

At selected time points, changes in the glutathione redox potential (GSH/GSSG) were evaluated using a GFP-based assay, namely Premo Cellular Redox Sensor (roGFP-Grx1) (P36242, Thermo Fisher Scientific, Waltham, MA, USA) as previously described.^34^ The glutathione redox potential is presented as a ratio of RFU_400 nm_ to RFU_488 nm_.

### 2.11 Biocompatibility Assay

To analyze the biocompatibility of MNP@CD26 and MNP@CD26@17D, *in vitro* hemolysis test was used as previously described.^32^ The erythrocyte lysis was assayed upon 24 and 48 h of incubation with MNP@CD26 and MNP@CD26@17D (30 and 60 μg of Fe/mL). As a positive control (PC), a treatment with 37.5 mM KCl was applied. Hemolysis in KCl-treated samples is considered as 100%.

### 2.12 Analysis of Gene Mutation

Data sets of gene mutation status in A431 skin cancer cells were acquired from the Dependency Map (DepMap) portal (https://depmap.org/portal/) (raw data are included in the Supporting Information). The selected variants were evaluated for gene enrichment in three databases, Reactome, KEGG, and GO Pathways, with the use of Kobas standalone software.^35^ The pathways associated with iron metabolism, autophagy, apoptosis, necroptosis, surface receptors, and oxidative stress and those related to cancer were further analyzed. Gene mutation types were presented using https://www.bioinformatics.com.cn as a chord diagram.

### 2.13 Statistical Analysis

The data are presented as mean ± standard deviation. Three biological replicates were considered. Box and whisker plots with median, lowest, and highest values were also used. Statistically significant differences between control and treated samples were evaluated using one-way analysis of variance (ANOVA) and Dunnett’s multiple comparison test using the GraphPad Prism 8 software. *P* values lower than 0.05 were assumed as statistically significant.

## 3 Results and Discussion

### 3.1 Levels of CD26 Are Increased in Senescent Fibroblasts and Skin Cancer Cells

It is widely accepted that the elimination of senescent cells can be improved by the use of antibody-based targeted senolytics.^13,14^ In this regard, a plethora of senescence-associated cell surface markers that could be potentially targeted have been already proposed.^17,36,37^ However, limited components of the senescent surfaceome were comprehensively and functionally characterized and validated as universal cell surface markers in numerous senescent cell types.

**Figure 1:**
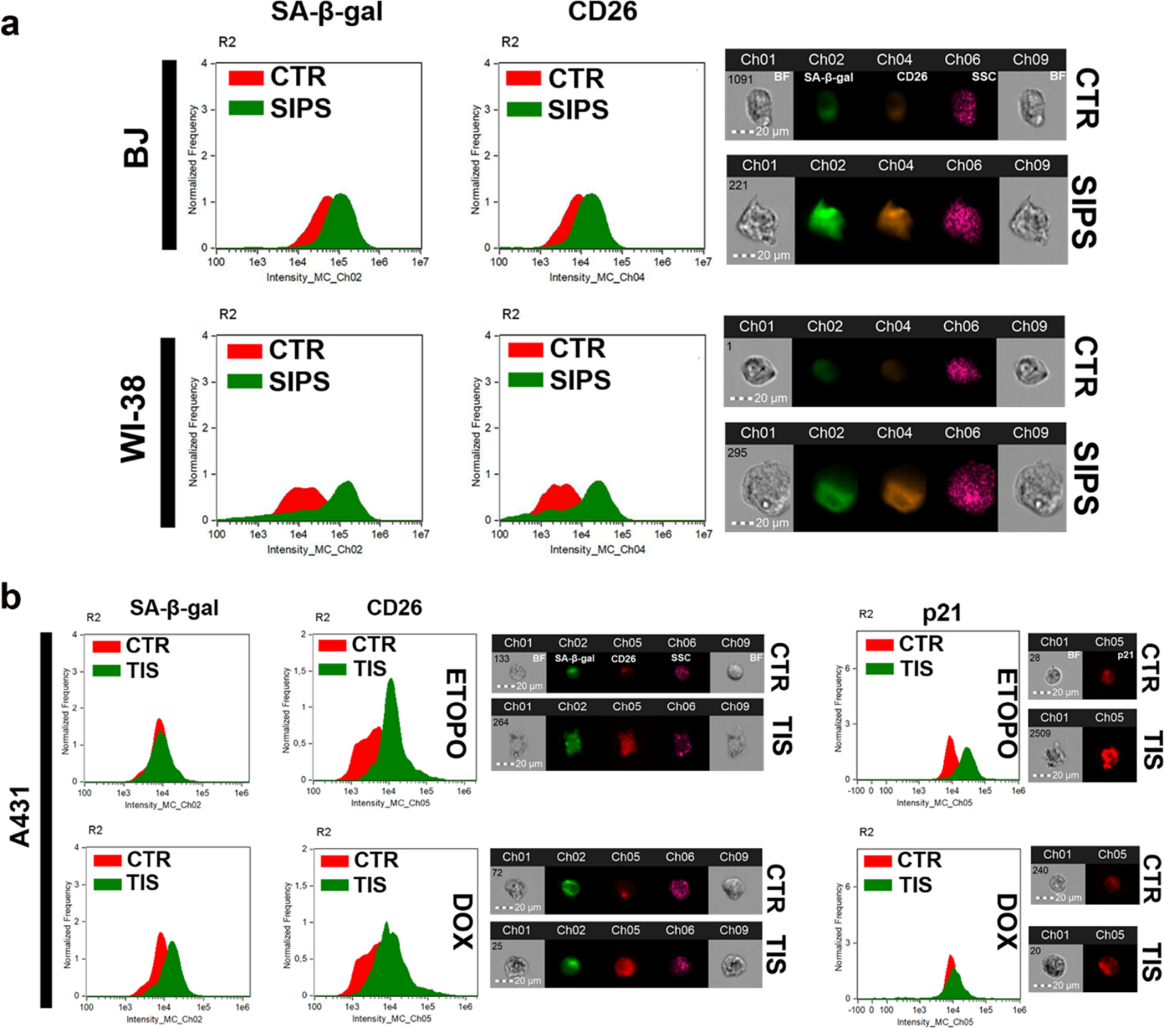
CD26 (DPP4) is a biomarker in (a) senescent normal fibroblasts (BJ and WI-38 cells) and (b) skin cancer A431 cells. The levels of CD26 in hydrogen peroxide-induced senescent fibroblasts (cellular model of stress-induced premature senescence, SIPS) and etoposide- and doxorubicin-induced senescent skin cancer cells (cellular models of therapy-induced senescence, TIS) and nonsenescent cells (CTR) were analyzed using imaging flow cytometry and a dedicated anti-CD26 antibody. Two hallmarks of cellular senescence were also assayed for comparison, namely, senescence-associated beta-galactosidase (SA-β-gal) activity and the levels of the cell cycle inhibitor p21. Representative histograms (green histograms, senescent cells; red histograms, nonsenescent cells) and microphotographs (Ch01 and Ch09, bright field (BF); Ch02, SA-β-gal activity; Ch04 (fibroblasts) or Ch05 (skin cancer cells), CD26; Ch06, side scatter, SSC) are presented. To evaluate the levels of p21, Ch05 was also used. R2, gated single cell population.

We were inspired by the work of Kim et al.^21^ who documented in 2017 that CD26 was selectively expressed on the surface of senescent, but not proliferating, human diploid fibroblasts. More recently, increased levels of CD26 were also reported in senescent mesenchymal stromal cells,^38^ vascular smooth muscle cells,^39^ and various lung cell types.^40^ However, there are no data reporting whether the levels of CD26 can also be affected in cancer cells upon induction of a senescence program by chemotherapeutic treatment and whether CD26 may also be a senescence marker in cancer cells. Thus, in the present study, we have evaluated and compared the pools of CD26 in oxidative stress-induced senescent human fibroblasts (BJ and WI-38), in anticancer drug-induced senescent A431 skin cancer cells, and corresponding nonsenescent cells (Figure 1).

To induce the senescence program, normal cells were challenged with an oxidant hydrogen peroxide (stress-induced premature senescence, SIPS) and skin cancer cells were stimulated with two anticancer drugs, namely, doxorubicin and etoposide (therapy-induced senescence, TIS).^31,33^ This approach would allow one to document whether CD26 levels are affected in different senescent cell types using different senescence-inducing conditions and to test whether CD26 can be considered as a more common or more private marker of senescence (universal or unique marker of senescence, respectively). The development of the senescence phenotype was monitored using imaging flow cytometry and the senescence-associated beta-galactosidase (SA-β-gal) activity test (Figure 1). Increased SA-β-gal activity was correlated with elevated levels of CD26 in senescent BJ skin and WI-38 lung fibroblasts; however, the effects were more pronounced in WI-38 cells (Figure 1a) as previously reported.^21^

Before the evaluation of CD26 levels in drug-induced senescent skin cancer A431 cells, we considered the bioinformatic analysis of gene mutations in surface receptors and related pathways (Figure S1). Different types of gene mutations were revealed in surface receptor-related genes, namely, in-frame deletions, missense, nonsense, nonstop, and silent mutations, but no mutations were identified in the gene encoding for CD26 (Figure S1). Thus, one can conclude that the putative function of CD26 is maintained and may be targetable in these cells. As TIS may be more heterogeneous when activated in cancer cells, two models of TIS (doxorubicin and etoposide treatments) and two markers of senescence (SA-β-gal activity, p21 levels) were considered in senescent skin cancer cells (Figure 1b). In the case of etoposide-induced senescence, the levels of a cell cycle inhibitor p21 served as a better biomarker of senescence than SA-β-gal activity compared with doxorubicin-mediated senescence in A431 cells (Figure 1b). Using two models of TIS, elevated levels of CD26 were revealed in drug-stimulated senescent cancer cells (Figure 1b). As the levels of CD26 were increased more in etoposide-induced senescent skin cancer cells than in doxorubicin-induced senescent skin cancer cells (Figure 1b), the former was selected for further analysis.

### 3.2 Design and Characterization of the Magnetic Nanoplatform for Target Senescent Cells

**Scheme 1:**
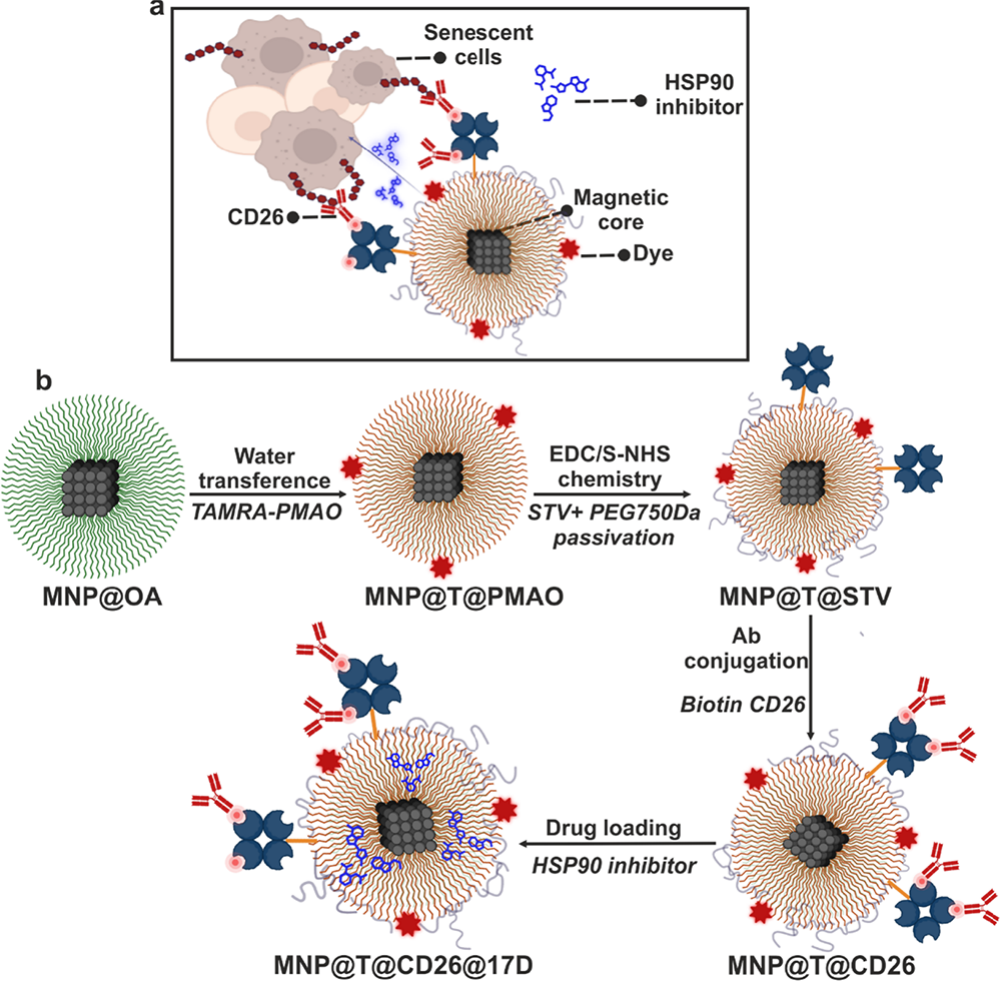
MNP@T@CD26@17D Nanoplatform Designed to Target and Eliminate Senescent Cells and Step-by-Step Process of MNP@T@CD26@17D Design (a) Schematic representation of MNP@T@CD26@17D nanoplatform designed to target and eliminate senescent cells. (b) Step-by-step process of MNP@T@CD26@17D design comprising (i) water transference by PMAO polymer addition (MNP@T@PMAO), (ii) functionalization with streptavidin (STV) by EDC/S-NHS chemistry and passivation of the MNP surface using PEG 750 Da (MNP@T@STV), (iii) bioconjugation with biotin modified CD26 antibody (MNP@T@CD26), and (iv) drug loading (17-DMAG HSP90 inhibitor) (MNP@T@CD26@17D).

As these results suggested that CD26 could be considered as a senescence biomarker in normal and skin cancer cells (Figure 1), we then used an anti-CD26 antibody and a nanobased approach to detect and target senescent cells. Furthermore, to potentiate anti-CD26 antibody-mediated cytotoxicity,^21^ two HSP90 inhibitors 17-AAG (tanespimycin) and 17-DMAG (alvespimycin) with senolytic activity^41^ were loaded. Briefly, the nanoplatform developed for targeting and killing senescent cells consisted of cubic iron oxide nanoparticles (MNPs) coated with a polymer poly(maleic anhydride-*alt*-1-octadecene) (PMAO) that was previously modified with a dye to track MNPs inside cells (TAMRA, T). These MNPs were functionalized with streptavidin (STV) to subsequently attach a biotin-modified CD26 antibody in order to target senescent cells (Scheme 1). Lastly, the nanoplatform was also loaded with HSP90 inhibitors, either 17-AAG (MNP@CD26@17A) or 17-DMAG (MNP@CD26@17D).

**Figure 2:**
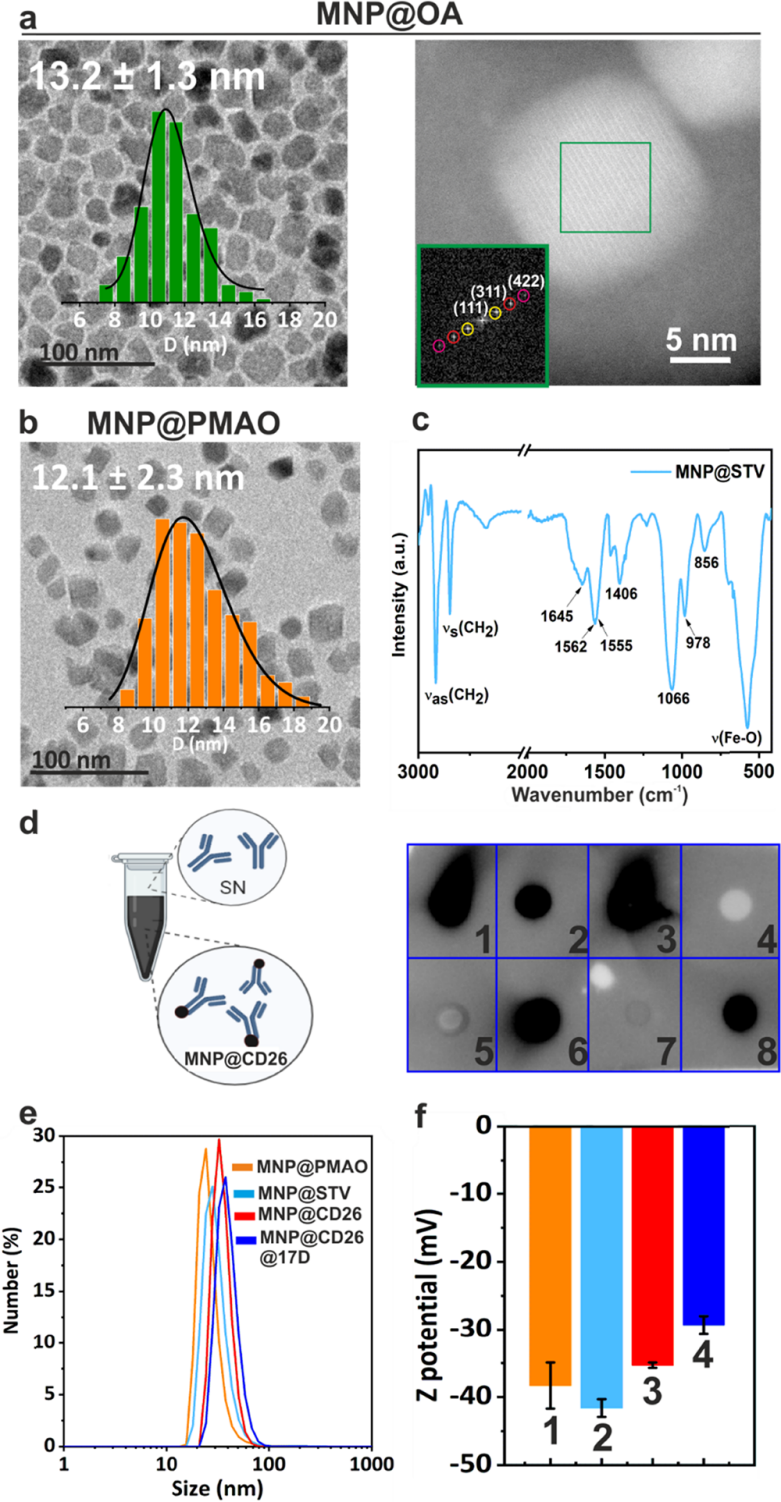
Physico-chemical characterization of the nanoplatform. (a) Left: TEM micrograph of as-synthesized MNP@OA and its size distribution (13.2 ± 1.3 nm). Right: high-resolution bright field STEM image of MNP@OA and the corresponding FFT diffraction showing the crystal planes. (b) TEM image of MNP transferred into water using PMAO (MNP@PMAO) and size distribution (12.1 ± 2.3 nm). (c) FT-IR spectra of STV functionalized MNP (MNP@STV). (d) Scheme of the quantification of unbound Ab present on the supernatants by dot-blot after their functionalization on MNP@STV. Semiquantitative dot-blot of the supernatants (SN) and the MNPs once functionalized with Ab. (1) Anti-CD26 (10 μg); (2) SN MNP@Ab (10 μg); (3) MNP@Ab (10 μg) (4–5) MNP@STV; (6) Anti-CD26 (2.5 μg); (7) SN MNP@Ab (2.5 μg); (8) MNP@Ab (2.5 μg). (e) Hydrodynamic diameters from DLS (by number) of MNP@PMAO, MNP@STV, MNP@CD26, and MNP@CD26@17D. (f) ζ-potential of the MNPs obtained after each step of functionalization labeled as (1) MNP@PMAO, (2) MNP@STV, (3) MNP@CD26, and (4) MNP@CD26@17D.

Figure 2 summarizes the main physicochemical characteristics of the MNPs before and after each functionalization step. MNPs were synthesized by one-step thermal decomposition, obtaining MNPs coated with oleic acid (MNP@OA).^24^

TEM images showed an anisometric cubic-like shape and an average size of 13.2 ± 1.3 nm of the MNP@OA magnetic core (Figure 2a). The resulting Fourier transformation of the high resolution-STEM image (right panel of Figure 2a) gave a pattern typical of single crystals in which it is possible to identify several planes like the (111), (311), and (422), which are indexed with the interplanar distances of a cubic spinel crystalline phase. The hydrophobic MNP@OA MNPs were transferred into the water using an amphiphilic polymer, PMAO, that renders MNPs soluble in water and provides them with carboxylic groups for further functionalization.^25^ As shown in Figure 2b, the shape and size (12.1 ± 2.3 nm) of the MNPs were maintained after polymer coating (MNP@PMAO). Magnetic properties of MNP@PMAO were evaluated by recording the hysteresis loops at 300 and 5 K. The MNPs showed a superparamagnetic behavior at room temperature with negligible coercivity or remanence (Figure S2). Magnetization values were normalized to the organic content measured by TGA (Figure S3) and all magnetic parameters are presented in Table S1.

We confirmed the successful coating of the core with PMAO using TGA and FT-IR spectroscopy (Figures S3 and S4). The increase of the organic content from 10% (MNP@OA) up to 32% (MNP@PMAO) is in agreement with the incorporation of the polymer shell.^42^ FT-IR analysis revealed a decrease of the stretching vibration of CH_2_ groups from the oleic acid and a shift to lower frequency of the _v_(COO^–^) mode indicating different coordination geometries as a result of PMAO addition (Figure S4). The iron phase (_V_Fe–O) was retained after water transference.

The next step was to functionalize the nanoplatform with CD26 Ab to target senescent cells. It is well-known that the orientation of the Ab on the MNP surface is critical to obtaining a better recognition of the antigen.^43,44^ Among the different methodologies that can be used to orient the Ab on the MNP, we selected specific adaptor molecules. Among them, STV is one of the best well-known adaptor proteins due to its high specificity and high interaction strength with biotin. Moreover, the interaction between STV and biotin is the strongest among the noncovalent bonds, being resistant to changes in pH, temperature, or ionic strength.^45^ To bind the STV to the MNPs, the carboxylic groups of the MNPs were activated by using carbodiimide (EDC)/*N*-hydroxysulfosuccinimide (S-NHS) chemistry. Afterward, the remaining activated carboxylic groups on the MNP surface were passivated with PEG 750 Da. The FT-IR spectrum of the resulting MNP@STV showed a new intense peak at 1066 cm^–1^ corresponding to −C-O vibration and the peaks at 978 cm^–1^ which can be assigned to C–H rocking in the PEG chain. Moreover, the C=O stretching vibration accounting for the amide I bond formed between the amino groups of STV with the free carboxyl moieties of the MNP@PMAO was observed (Figure 2c). Analysis of the supernatant by the Bradford assay revealed that all the STV that was being added was coupled to the MNPs (not shown). We estimated that the amount of conjugated STV was six molecules for each MNP (Section S1 in the Supporting Information).

We then added different amounts of CD26 Ab (modified with biotin) to these MNPs to decide which was the best ratio to use. The resulting MNPs (MNP@CD26) were centrifuged, and the supernatants of the functionalization were conserved and quantified using a semiquantitative dot blot. Considering that the supernatants contain the unbound Ab, the signal obtained in this assay will be inversely proportional to the amount of Ab attached to the MNPs^42^ (Figure 2d). As controls, samples containing the same amounts of Abs used to functionalize the MNPs were utilized (100% added Ab). As shown in Figure 2d when 10 μg of Ab (per 0.3 mg of Fe) was used, a strong signal could be obtained in the supernatant, indicating that not all the added Ab was bound to the MNPs. However, when 2.5 μg was used, the supernatant did not give any signal, indicating complete binding of the Abs to the MNPs. When instead of the supernatants, the MNP@CD26 were analyzed, a strong signal could also be observed, confirming the presence of Ab on the MNPs surface. As expected, when MNP@PMAO and MNP@STV (without Ab) were analyzed, no visual signal was obtained.

The last step was to incorporate the drug into the nanoplatform. Given that the MNP core is coated with oleic acid, drugs can be adsorbed between these molecules and the hydrophobic tails of the PMAO. Different amounts of 17-AAG or 17-DMAG were added to the MNP@CD26 (MNP@CD26@17A and MNP@CD26@17D, respectively), finding that high amounts of both drugs (60 nmol/0.1 mg of Fe) led to the irreversible aggregation of the MNPs. When 30 nmol/0.1 mg of Fe (17-AAG) or 20 nmol/0.1 mg of Fe (17-DMAG) was used, the MNPs remained stable over time. The loading efficiency was determined indirectly by measuring the amount of unbound drug in the supernatant by using a spectrophotometer. For both drugs 17-AAG and 17-DMAG, the loading efficiency was about 30%. Figure 2e shows the hydrodynamic size distributions of the MNPs after each functionalization step. Hydrodynamic sizes slightly increased when adding the different components of the nanoplatform, which could be indicative of a successful functionalization. Regarding superficial charge, all MNPs showed a negative ζ-potential because of the carboxylic groups present on the PMAO (Figure 2f).

At this point, we initially evaluated the antioxidant/pro-oxidant properties of MNPs in a cell-free micellar system *in vitro*. The rate of peroxidation of a lipid emulsion without additives, blank sample (Table S2 and Figure S5) was compared with the rate of peroxidation in the presence of: MNP@PMAO, 17-DMAG free drug, MNP@CD26, and MNP@CD26@17D. In this experimental system, the additives were kinetically neutral as they did not produce any significant inhibition or acceleration of peroxidation (within experimental error; see last column in Table S2).

### 3.3 MNP@CD26@17D Displays Senolytic Activity against Senescent Normal and Cancer Cells

It has been reported that the HSP90 inhibitor 17-DMAG, when used at a concentration of 100 nM for 6 and 24 h, may overcome apoptosis resistance in senescent mouse embryonic fibroblasts (MEFs) by decreasing the activity of a pro-survival kinase Akt, a HSP90 client protein.^41^ Interestingly, the senolytic activity of HSP90 inhibitors was not cell type-specific or species-specific, as HSP90 inhibitors used at the nanomolar range, were active against senescent MEFs, human lung fibroblasts (IMR90 and WI-38), and vascular endothelial cells (HUVECs).^41^ The senolytic activity of HSP90 inhibitors was also validated in different models of cellular senescence, such as oxidative stress, genotoxic stress, and replicative stress-driven senescence.^41^ As we have already mentioned, systemic administration of senolytics may promote some off-target side effects.^6^ Thus, it is reasonable to maximize the efficacy and limit the adverse effects of senolytics using targeted senolytics, especially nanosenolytics.^13,14^ In the present study, the senolytic action of a multifunctional nanoplatform containing MNP@CD26 and the senolytic drugs 17-AAG or 17-DMAG was tested against oxidant-induced senescent human fibroblasts (BJ and WI-38) and drug-induced senescent skin cancer cells (A431).

**Figure 3:**
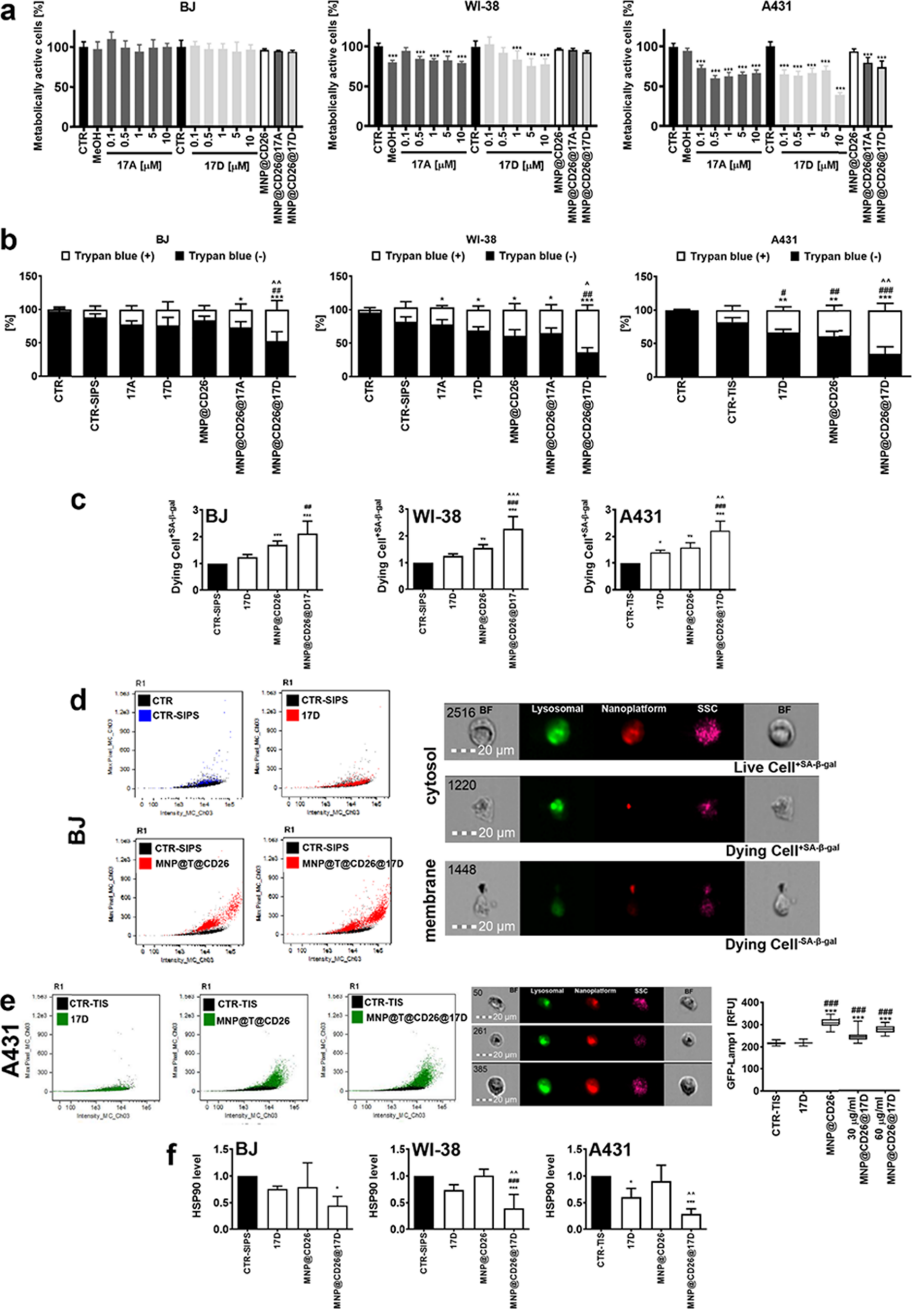
Effects of drugs and nanoplatform on nonsenescent cells and MNP@CD26@17D stimulated necrosis-based senolysis, necrotic morphotype of cell death, in senescent normal and cancer cells. (a) Nonsenescent normal (BJ, WI-38) and cancer (A431) cells were treated with the drugs 17-AAG and 17-DMAG in a range of concentrations starting from 0.1 to 10 μM or the nanoplatforms (60 μg of Fe/mL) for 24 h, and metabolic activity was then assayed using MTT test. Metabolic activity at control untreated conditions (CTR) is considered as 100%. The effects of the 17-AAG vehicle, namely methanol (MeOH) are also presented. There were no effects of distilled water, a solvent used for 17-DMAG (data not shown). (b–f) Senescence program was induced in normal and cancer cells by hydrogen peroxide (SIPS) and etoposide (TIS) stimulation, respectively. (b) Necrosis (porous cell membrane) was analyzed using trypan blue staining and inverted light microscopy. (c) Imaging flow cytometry-based analysis of dying cells (necrotic morphotype of cell death) with senescence-associated beta-galactosidase (SA-β-gal) activity. (d, e) MNP@CD26@17D uptake and cellular localization. For uptake analysis, nanoplatform containing TAMRA was used (red). TAMRA-based signals were colocalized with SA-β-gal-based signals (green) that confirms MNP@CD26@17D uptake and lysosomal localization. Representative dot plots and microphotographs are shown. The analysis of the levels of lysosomal marker Lamp1 was also considered using GFP-Lamp1 fusion protein and confocal imaging. GFP-Lamp1 signals are presented as relative fluorescence units (RFU). (f) MNP@CD26@17D-mediated necrosis-based senolysis is accompanied by decreased levels of HSP90. HSP90 levels were assayed using imaging flow cytometry and a dedicated anti-HSP90 antibody. Bars indicate SD or box and whisker plots are shown, *n* = 3, ***p < 0.001, **p < 0.01, *p < 0.05 compared to control (a, b) or senescence control (c, e, f) (ANOVA and Dunnett’s a posteriori test), (b) ^###^*p* < 0.001, ^##^*p* < 0.01, ^#^*p* < 0.05 compared to senescence control (ANOVA and Dunnett’s a posteriori test), ^ ^*p* < 0.01, ^*p* < 0.05 compared to 17-DMAG alone (ANOVA and Tukey’s a posteriori test), (c, e, f) ^###^*p* < 0.001, ^##^*p* < 0.01 compared to 17-DMAG alone (ANOVA and Tukey’s a posteriori test), ^ ^ ^*p* < 0.001, ^ ^*p* < 0.01 compared to MNP@CD26 (ANOVA and Tukey’s a posteriori test). SIPS, stress-induced premature senescence; TIS, therapy-induced senescence; 17A, 17-AAG treatment; 17D, 17-DMAG treatment; MNP@CD26, nanoplatform containing anti-CD26 antibody; MNP@CD26@17A, nanoplatform containing anti-CD26 antibody and 17-AAG; MNP@CD26@17D, nanoplatform containing anti-CD26 antibody and 17-DMAG; MNP@T@CD26, nanoplatform containing TAMRA dye and anti-CD26 antibody; MNP@T@CD26@17D, nanoplatform containing TAMRA dye, anti-CD26 antibody, and 17-DMAG. R1, gated single cell population.

Initially, the effects of free drugs in a range of concentrations (0.1 to 10 μM) were tested in nonsenescent cells using the MTT assay (Figure 3a). We have included the senolysis-promoting concentration (100 nM) of drugs^41^ to reveal their effects on nonsenescent cells (Figure 3a). In general, the drugs did not affect the metabolic activity of BJ fibroblasts, even when higher concentrations were used (Figure 3a). Conversely, WI-38 fibroblasts were slightly affected when drugs were used at high concentrations (Figure 3a). Surprisingly, A431 skin cancer cells were the most sensitive to drug treatment (Figure 3a).

The senolysis-promoting concentration (100 nM) of 17-AAG and 17-DMAG^41^ decreased the metabolic activity of A431 cells to 73 and 65% of control levels, respectively, whereas the metabolic activity of BJ and WI-38 fibroblasts was not affected (Figure 3a). For the highest concentration considered (10 μM), 17-DMAG-mediated inhibitory activity was much more pronounced than 17-AAG-mediated inhibitory activity against A431 skin cancer cells (Figure 3a). It should be highlighted that the senolytic activity of HSP90 inhibitors was documented against senescent normal cells and their effects against senescent cancer cells were not established.^41^ One can speculate that senescent cancer cells might respond differently than senescent normal cells to the treatment with HSP90 inhibitors that we have observed for nonsenescent normal versus cancer cells (Figure 3a).

On the other side, the nanoplatforms (MNP@CD26, MNP@CD26@17A, and MNP@CD26@17D), when used at the concentration of 60 μg of Fe/mL, did not affect the metabolic activity of normal cells (BJ and WI-38 cells), whereas the nanoplatforms loaded with drugs 17-AAG and 17-DMAG promoted a decrease in the metabolic activity of A431 skin cancer cells of about 20 and 26% compared to untreated control, respectively (Figure 3a).

The amount of nanoplatform used produced effects similar to those of 100 nM free drugs. So, for further analysis of the senolytic activity of the nanoplatforms against senescent normal and cancer cells, the effects of this nanoplatform concentration were compared with the effects of free drugs at the previously reported concentration of 100 nM.^41^

First, trypan blue staining was used to document the necrosis-based senolytic action of free drugs alone, MNP@CD26, and MNP@CD26@17A or MNP@CD26@17D in senescent fibroblasts upon 24 h of treatment (Figure 3b). The effects of senolytic drugs alone were mild to moderate, and the addition of 17-DMAG as a component of functionalized nanoplatform significantly potentiated necrosis-based morphotypes of cell death in both fibroblast cell lines (Figure 3b). As MNP@CD26@17A did not produce this effect in normal senescent cells, only MNP@CD26@17D was selected for further studies (Figure 3b). Moreover, MNP@CD26@17D promoted massive necrotic cell death in drug-induced senescent A431 skin cancer cells compared with the action of free 17-DMAG (Figure 3b).

We then combined imaging flow cytometry and SA-β-gal staining to document that upon MNP@CD26@17D treatment, the major fraction of dying cells was the fraction of SA-β-gal-positive cells (senescent cells) (Figure 3c). Thanks to the fluorophore that was incorporated in the PMAO coating, MNP@T@CD26@17D uptake was also documented by using imaging flow cytometry (Figure 3d,e). The nanoplatform was accumulated in the lysosomal compartment, as judged by the colocalization analysis of lysosomal SA-β-gal activity (Figure 3d). Increased levels of lysosomes were also observed upon MNP@CD26 and MNP@CD26@17D treatment using the analysis of fluorescence signals of lysosomal marker Lamp1 fused to green fluorescence protein (GFP) (Figure 3e). This result confirms that the nanoplatform is indeed taken up by senescent cells. Interestingly, MNP@CD26@17D-treated senescent cells were also characterized by lower levels of HSP90 compared to 17-DMAG-treated senescent cells as judged by immunofluorescence-based analysis of HSP90 pools (Figure 3f). Thus, one can conclude that MNP@CD26@17D-mediated senolytic action is associated with decreased pools of the pro-survival factor HSP90 (Figure 3f).

To improve the senolytic effects of the nanoplatform, the possibility of increasing the incubation time was considered. Before that, a simple hemolysis-based hemocompatibility test was performed to initially assess the biocompatibility of MNP@CD26@17D, when used at concentrations of 30 and 60 μg of Fe/mL for 24 and 48 h. MNP@CD26@17D did not promote the hemolysis of human erythrocytes upon 24 stimulation (Figure S6). However, prolonged treatment (48 h stimulation) decreased the viability of red blood cells (Figure S6). Thus, 24 h of incubation was the maximum time selected for further experiments.

### 3.4 Dual Response to MNP@CD26@17D Stimulation in Senescent Cells

**Figure 4:**
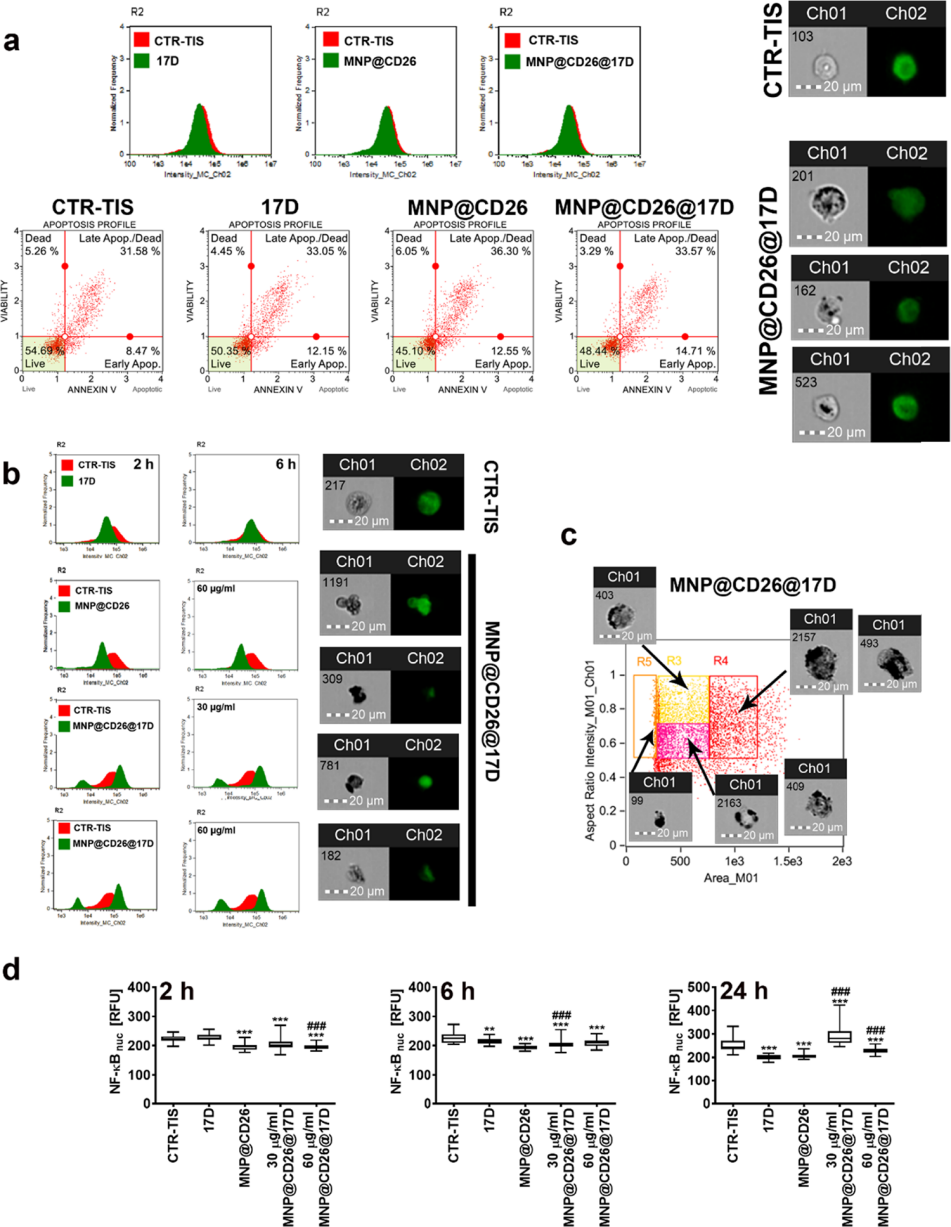
MNP@CD26@17D-promoted apoptosis in drug-induced senescent A431 skin cancer cells. (a, b) Three time points (2, 6, and/or 24 h) and two markers of apoptosis were considered, namely, caspase 3 levels (imaging flow cytometry using a dedicated anticaspase 3 antibody, Ch02, green signals) and phosphatidylserine externalization (flow cytometry and Annexin V staining). (a) Analysis of cellular apoptosis (caspase 3 levels and Annexin V staining) after 24 h of treatment with 17-DMAG, MNP@CD26, and MNP@CD26@17D. (b) Short-term treatment with MNP@CD26@17D (2 and 6 h) resulted in apoptosis induction (apoptosis-based senolytic effect) as seen by analyzing caspase 3 levels. Representative histograms, dot plots, and microphotographs are shown. (c) MNP@CD26@17D treatment, if prolonged, promoted cell fragmentation and cellular, here, morphological, heterogeneity. Cell morphology was assayed using imaging flow cytometry. Representative morphological features within cell subpopulations are presented (arrows). (d) Decreased nuclear levels of NF-κB, a transcription factor with antiapoptotic function upon short-term stimulation with the nanoplatform. Results are presented as relative fluorescence units (RFU). Box and whisker plots are shown, *n* = 3, ***p < 0.001, **p < 0.01 compared to senescence control (ANOVA and Dunnett’s a posteriori test), ^###^*p* < 0.001 compared to 17-DMAG alone (ANOVA and Tukey’s a posteriori test). TIS, therapy-induced senescence; 17D, 17-DMAG treatment; MNP@CD26, nanoplatform containing anti-CD26 antibody; MNP@CD26@17D, nanoplatform containing anti-CD26 antibody and 17-DMAG. R2, gated single cell population.

As first-generation senolytics were reported to induce cell death in senescent cells by means of apoptotic cell death,^8^ MNP@CD26@17D-mediated apoptosis was then assayed in senescent skin cancer cells upon 24 h of treatment (Figure 4a). Surprisingly, there were no statistically significant increases in the levels of two markers of apoptosis, namely, phosphatidylserine externalization or caspase 3 pools in MNP@CD26@17D-treated senescent skin cancer cells (Figure 4a). Similar results were also observed in senescent fibroblasts (Figure S7a,b). Thus, we decided to analyze an earlier response, namely, upon 2 and 6 h of stimulation with MNP@CD26@17D (Figure 4b). We mainly focused on drug-induced senescent skin cancer cells as the effects of CD26 targeting were initially established in senescent normal cells, but no data are available regarding senescent cancer cells.^21^

In this case, an increase in caspase 3-positive cells was observed in MNP@CD26@17D-treated senescent skin cancer cells at both concentrations of 30 and 60 μg/mL of Fe but not in 17-DMAG or MNP@CD26-treated cells (Figure 4b). However, a fraction of caspase 3-positive cells with lower fluorescence intensity and smaller cell size also occurred, suggesting that the apoptosis-based early response to MNP@CD26@17D treatment was accompanied by a massive cell fragmentation (Figure 4b). Based on these results, we then conducted a more comprehensive analysis of the morphological features of MNP@CD26@17D-treated senescent skin cancer cells using imaging flow cytometry (Figure 4c). Indeed, a fraction of small fragmented cells and cells with damaged cell membranes and affected cell morphology were noticed (Figure 4c), confirming that 2 or 6 h of treatment with MNP@CD26@17D caused cell fragmentation (Figure 4c). As nuclear factor-kappa B (NF-κB), a multifunctional transcription factor, can be also considered as a prosurvival and antiapoptotic factor upregulating antiapoptotic genes and promoting the drug resistance in cancer cells,^46,47^ we decided then to analyze the nuclear pools of NF-κB upon short versus long-term treatment with the nanoplatform (Figure 4d). The levels of NF-κB were decreased after 2 and 6 h of treatment with the nanoplatform, suggesting that at these time points, senescent skin cancer cells cannot be protected against nanoplatform-mediated apoptosis by NF-κB transcriptional activity (Figure 4d). However, upon 24 h stimulation with 30 μg/mL MNP@CD26@17D, the nuclear levels of NF-κB were increased, explaining at least in part why apoptotic cell death was not observed upon long-term treatment (Figure 4a).

We have then analyzed the status of gene mutations in different functional gene categories in A431 cells and found that the group of apoptosis-related genes was characterized by the highest number of gene mutations among the groups considered (Figure S8). Furthermore, to predict the response of A431 cells to apoptotic stimuli, a comprehensive analysis of gene mutations in genes related to apoptosis was conducted (Figure S8). The mutation (in frame deletion) in the gene encoding for caspase 8 was identified that may explain mild to moderate apoptotic response upon MNP@CD26@17D stimulation in A431 cells (Figure S8). As 24 h of treatment with MNP@CD26@17D resulted in a necrotic morphotype of cell death, one can also conclude that a switch between apoptosis and necrosis may be observed. Of course, it is worthwhile investigating if this necrotic cell death is of an unregulated or regulated nature, i.e. ferroptosis.^48^

**Figure 5:**
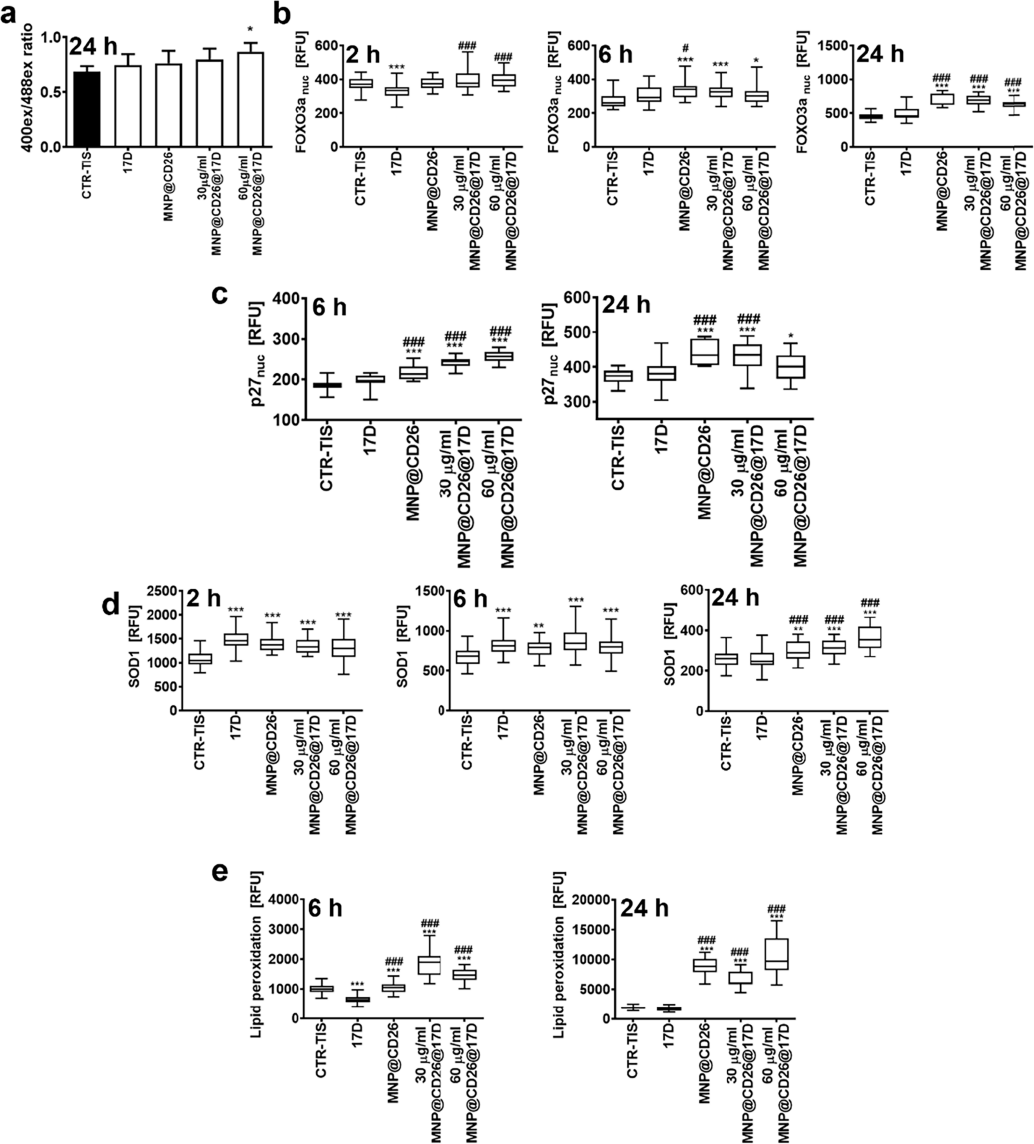
MNP@CD26@17D-associated redox imbalance in drug-induced senescent A431 skin cancer cells. Three time points (2, 6, and/or 24 h) were considered. (a) Glutathione redox potential (GSH/GSSG) was assayed as an oxidation status of a redox sensor Grx1-GFP using fluorescence mode microplate reader. Results are presented as a ratio of 400_ex_ to 488_ex_. Bars indicate SD, *n* = 3, *p < 0.05 compared to senescence control (ANOVA and Dunnett’s a posteriori test). (b–d) Imaging cytometry-based analysis of nuclear levels of FOXO3a and p27, and total levels of SOD1 using dedicated antibodies. (e) Imaging cytometry-based analysis of lipid peroxidation using dedicated kit. (b–e) Results are presented as relative fluorescence units (RFU). Box and whisker plots are shown, *n* = 3, ***p < 0.001, **p < 0.01, *p < 0.05 compared to senescence control (ANOVA and Dunnett’s a posteriori test), ^###^*p* < 0.001, ^#^*p* < 0.05 compared to 17-DMAG alone. TIS, therapy-induced senescence; 17D, 17-DMAG treatment; MNP@CD26, nanoplatform containing anti-CD26 antibody; MNP@CD26@17D, nanoplatform containing anti-CD26 antibody and 17-DMAG.

As different types of regulated cell death may be triggered or accompanied by oxidative stress,^48^ selected oxidative stress parameters were then studied (Figure 5).

The treatment with MNP@CD26@17D at 60 μg of Fe/mL for 24 h promoted a slight intracellular redox imbalance, as judged by the oxidation of the redox-sensitive biosensor Grx1-GFP that reflects changes in the glutathione redox potential (GSH/GSSG) (Figure 5a). Next, three time points were considered (2, 6, and 24 h) to analyze more comprehensively the oxidative stress mediated by MNP@CD26@17D (Figure 5b–e). The activation of FOXO3a, a transcription factor modulating oxidative stress response and ROS-regulated processes,^49,50^ was observed upon 6 and 24 h treatment with MNP@CD26@17D (30 and 60 μg of Fe/mL) (Figure 5b). A similar effect was not noticed in the case of stimulation with the free drug 17-DMAG (Figure 5b). Of note, the MNP@CD26 without drug also caused an increase in the nuclear levels of FOXO3a (6 and 24 h), suggesting that MNP functionalized with CD26 has an effect per se (Figure 5b). As FOXO3a may also mediate cell cycle inhibition and cell death,^49,50^ this result may reflect antibody-related cytotoxic effects and, in turn, a FOXO3a-based response. Indeed, at the same time points, MNP@CD26@17D and MNP@CD26 also promoted an increase in the levels of the cell cycle inhibitor p27 that was not promoted by the drug alone; however, the effects of MNP@CD26@17D were more pronounced (Figure 5c). FOXO3a also stimulated a superoxide dismutase 1 (SOD1)-based adaptive antioxidant response upon MNP@CD26@17D and MNP@CD26 treatment at all time points. As an early response, SOD1 levels were also increased in 17-DMAG-treated senescent skin cancer cells, but this was not observed after 24 h treatment (Figure 5d). Similarly, FOXO3a activation and elevated levels of SOD1 were also documented after 6 h treatment with MNP@CD26@17D, but not 17-DMAG, in senescent fibroblasts (Figure S7c).

More recently, we have also documented that manganese–iron oxide MNPs by means of magneto-thermal stimulation may promote ROS-mediated adaptive response modulating tissue regeneration *in vivo*.^51^ Manganese–iron oxide MNPs affected ROS production, the levels and the activity of selected antioxidant enzymes and oxidative stress-related transcription factors such as FOXO tuning regeneration dynamic *in vivo*.^51^

As MNP@CD26@17D promoted redox imbalance and accompanying oxidative stress response (Figure 5a,b,d), we were then interested in analyzing whether MNP@CD26@17D may also stimulate the accumulation of oxidatively damaged biomolecules, such as lipid peroxidation. MNP@CD26@17D, but not 17-DMAG, induced lipid peroxidation after 6 and 24 h of stimulation in senescent skin cancer cells (Figure 5e). This result inspired us to investigate if MNP@CD26@17D may promote lipid peroxidation-mediated cell death, namely ferroptosis in senescent cells, assumed as a type of regulated necrosis.^52,53^ As the analysis of gene mutations in genes regulating oxidative stress response revealed that a limited number of genes were mutated (Figure S9), we hypothesized that A431 cells could share similar sensitivity to oxidative stress as normal cells and could be susceptible to lipid peroxidation-mediated cytotoxicity.

**Figure 6:**
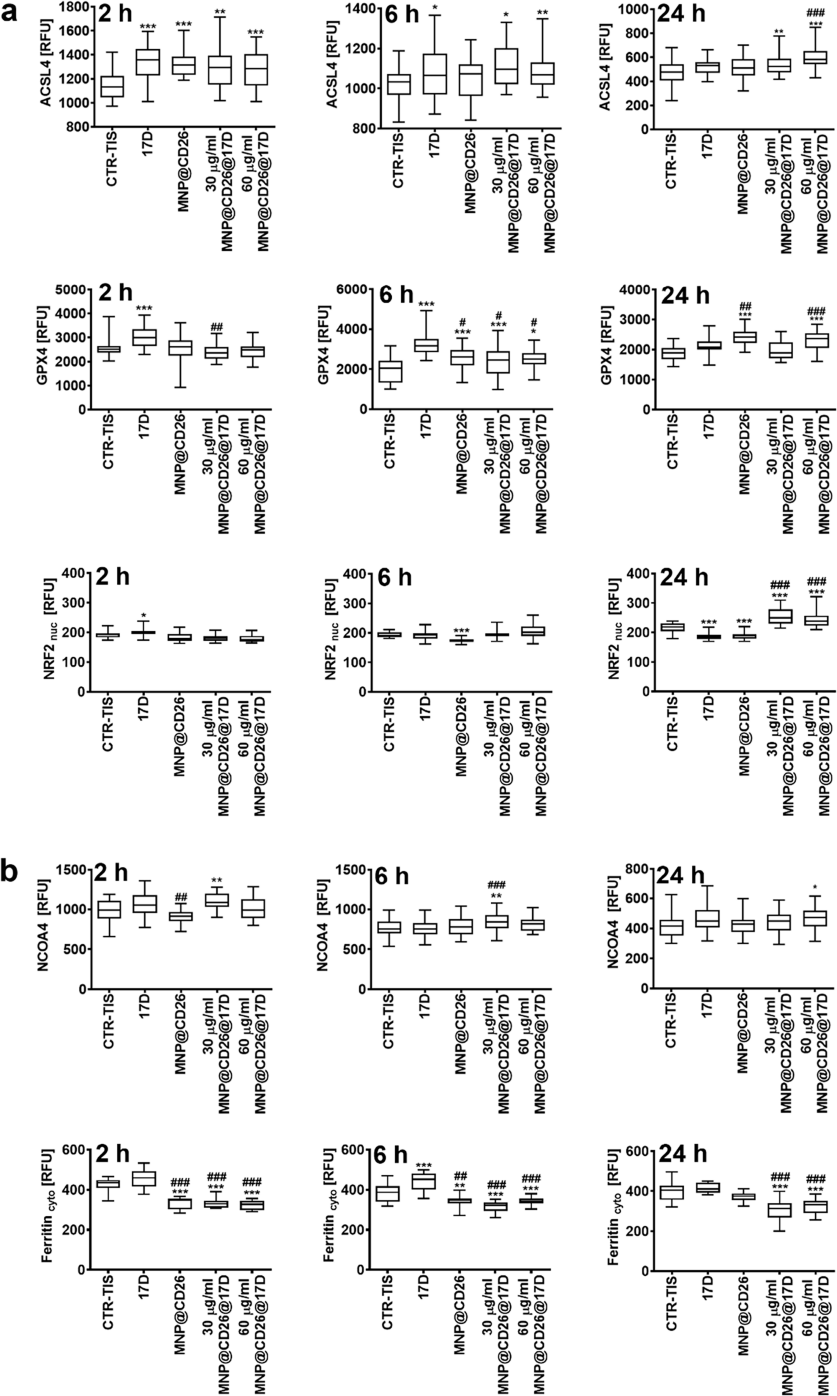
MNP@CD26@17D-mediated ferroptosis (a) and ferritinophagy (b) in drug-induced senescent A431 skin cancer cells. Three time points (2, 6, and/or 24 h) were considered. Ferroptotic (a, ACSL4, GPX4, and NRF2) and ferritinophagic (b, NCOA4, ferritin) markers were assayed using imaging cytometry and dedicated antibodies. The levels of the analyzed proteins are presented as relative fluorescence units (RFU). Box and whisker plots are shown, *n* = 3, ***p < 0.001, **p < 0.01, *p < 0.05 compared to senescence control (ANOVA and Dunnett’s a posteriori test), ^###^*p* < 0.001, ^##^*p* < 0.01, ^#^*p* < 0.05 compared to 17-DMAG alone. TIS, therapy-induced senescence; 17D, 17-DMAG treatment; MNP@CD26, nanoplatform containing anti-CD26 antibody; MNP@CD26@17D, nanoplatform containing anti-CD26 antibody and 17-DMAG.

Ferroptosis is an iron-dependent nonapoptotic cell death driven by lipid peroxidation of polyunsaturated fatty acids (PUFAs), structural components of membrane phospholipids.^53,54^ Lipid peroxidation, triggering ferroptotic cell death, might be initiated by acyl-coenzyme A (acyl-CoA) synthetase long-chain family member 4 (ACSL4)/ lysophosphatidylcholine acyltransferase 3 (LPCAT3)/ arachidonate 15-lipoxygenase (ALOX15)-dependent enzymatic reaction as well as Fe^2+^-dependent nonenzymatic Fenton reactions.^48^ As ACSL4, catalyzing the esterification of arachidonoyl or adrenoyl into phosphatidylethanolamines, is postulated to be a key factor of lipotoxicity in ferroptosis,^55,56^ the levels of ACSL4 were then assayed (Figure 6a). After 24 h of treatment with MNP@CD26@17D, the levels of ACSL4 were increased in senescent skin cancer cells (Figure 6a). The effect was not limited to only one senescence model *in vitro*, because in oxidant-induced senescent fibroblasts, ACSL4 pools were also elevated upon stimulation with MNP@CD26@17D (Figure S7c). Thus, MNP@CD26@17D may induce ferroptosis as a late senolytic response in senescent cells. Increased lipid peroxidation and ACSL4 levels were also accompanied by elevated levels of glutathione peroxidase 4 (GPX4) in cells treated with MNP@CD26@17D and MNP@CD26 for 6 and 24 h (Figure 6a).

This result is surprising as GPX4 is considered as a negative regulator of ferroptosis^57^ due to its ability to inhibit lipid peroxidation by reducing phospholipid hydroperoxides.^58^ However, under our experimental conditions, increased levels of GPX4 did not protect against MNP@CD26@17D-induced lipid peroxidation and ferroptotic cell death (Figure 6a). Perhaps GPX4 levels, along with SOD1 levels, were elevated as a result of the FOXO3a-mediated adaptive antioxidant response (Figures 5 and 6), but this was not sufficient to attenuate MNP@CD26@17D-associated cytotoxicity. Furthermore, cancer cells can adapt to oxidative stress by increasing the activity of the transcription factor nuclear factor erythroid 2-related factor 2 (NRF2) promoting tumorigenesis by the expression of numerous antioxidant proteins such as the regulators of GSH synthesis and metabolism, for example, GPX4.^59,60^ Thus, NRF2 is also considered a negative modulator of ferroptosis by GPX4-mediated attenuation of lipid peroxidation.^61^ Upon 24 h stimulation with MNP@CD26@17D, the nuclear levels of NRF2 were also increased in senescent skin cancer cells, which may also explain the elevated levels of GPX4 (Figure 6a); however, the increased transcriptional activity of NRF2 did not protect against MNP@CD26@17D-induced lipid peroxidation (Figure 5e) and ferroptosis. We suggest that even if elevated, GPX4 might not be fully functional, as a result of MNP@CD26@17D-mediated oxidative stress. To be an efficient protector against lipid peroxidation, GPX4 requires reduced glutathione (GSH) for the enzymatic reaction and must be then regenerated by means of GSH. Thus, if GSH pool is limited, GPX4 activity and GPX4-mediated protection against lipid peroxidation could also be limited. In our experimental conditions, MNP@CD26@17D treatment affected glutathione redox potential as judged by the increased oxidation status of the redox sensor Grx1-GFP (Figure 5a), suggesting a pro-oxidant shift in the glutathione redox state and the accumulation of the oxidized form of glutathione GSSG, affecting the functionality of GPX4. Furthermore, despite GPX4 being considered as a key regulator of ferroptosis, GPX4-independent ferroptosis pathways have been also established.^62^

Ferritinophagy is an autophagy process associated with ferroptosis, where ferritin, an iron storage protein, is degraded in autophagosomes mediated by nuclear receptor coactivator 4 (NCOA4).^63^ MNP@CD26@17D treatment in senescent skin cancer cells also resulted in increased levels of NCOA4 (Figure 6b) suggesting that MNP@CD26@17D-induced ferroptosis is stimulated by ferritinophagy. These data are supported by the fact that both short- and long-term treatment with MNP@CD26@17D resulted in a decrease in the pools of ferritin (Figure 6b) indicating that MNP@CD26@17D promotes the disequilibrium of iron homeostasis. Furthermore, 3% of iron release from MNPs after 2, 6, and 24 h of incubation in an artificial lysosomal medium was confirmed by ICP-OES (data not shown). This indicates that iron can be present over time in our *in vitro* system and after 24 h treatment released iron along with decreased levels of ferritin (Figure 6b) might promote ferritinophagy-mediated ferroptosis in senescent skin cancer cells.

We have previously demonstrated that similar MNPs (in shape and size and coated with the same polymer, PMAO) are able to enter the cell through endocytosis^26^ and that the MNPs can slowly degrade, both *in vitro* and *in vivo*.^64^ For instance, when MNPs are placed in the simulated lysosomal medium, they release iron ions due to their core degradation. If the degradation is high, then this phenomenon can be analyzed using transmission electron microscopy (TEM), as the size of the MNP decreases. Interestingly, we showed that MNPs could be degraded in a lysosomal medium but not in water.^64^ The dissolved iron ions were retained around the NP core as a ring,^64^ most probably forming coordination complexes with the carboxylic groups of the polymer.

It has been also reported that ferroptosis may be facilitated by transferrin receptor (TfR)-mediated iron accumulation in cancer cells.^65^ On the other hand, iron overload in senescent mice and human fibroblasts may also be accompanied by ferroptosis inhibition.^66^ Thus, iron accumulation may play a complex role during ferroptosis depending on the cellular context. Senescent normal cells were reported to have affected iron homeostasis mechanisms by potentiating both iron acquisition and sustained storage.^66^ Impaired ferritinophagy, an autophagic degradation of ferritin, resulted in functional cellular iron deficiency that, in turn, promoted TfR expression and iron accumulation in senescent cells.^66^ We have also observed that TfR levels were elevated in senescent skin cancer cells using the drug-induced senescence model (Figure S7d). Our data also agree with previously published results on the effects of activation of the autophagy pathway on ferroptosis induction by degradation of ferritin and increased labile iron pool in fibroblasts and cancer cells.^67^ Interestingly, it was also shown that CD26 may be involved in the regulation of ferroptosis in colorectal cancer cells with the loss of p53 (*TP53*^–/–^).^68^ It was documented that the interaction between CD26 and NADPH oxidase 1 (NOX1) is crucial for lipid peroxidation in ferroptosis in TP53-deficient (but not TP53-sufficient) colorectal cancer cells.^68^ Perhaps, in drug-induced senescent skin cancer A431 cells with elevated levels of CD26 (Figure 1b) and nonfunctional *TP53* gene (https://depmap.org/portal/cell_line/ACH-001328?tab=overview), CD26 may also modulate MNP@CD26@17D-induced ferroptosis. Furthermore, identified gene mutations in genes regulating iron metabolism in A431 cells may also affect MNP@CD26@17D-associated response (Figure S10). However, these issues require further investigation.

The activation of classical inflammatory signaling pathways such as the Janus kinase signal transducer and activator of transcription (JAK-STAT), NF-κB, inflammasome or cyclic GMP-AMP synthase stimulator of interferon genes (cGAS-STING) might be also implicated in ferroptotic cell death.^69^ Indeed, the activation of NF-κB upon 24 h of stimulation with 30 μg/mL MNP@CD26@17D was also observed in senescent skin cancer cells that were accompanied by ferroptosis induction (this study). In contrast, short-term treatment with the nanoplatform did not result in elevated levels of nuclear NF-κB and ferroptosis induction (this study). Thus, perhaps NF-κB might mediate the MNP@CD26@17D-based ferroptotic response in senescent skin cancer cells, but more evidence is needed to confirm such assumptions.

Heat shock proteins, a family of conserved molecular chaperones highly expressed under a plethora of stress conditions, may confer resistance to different modes of cell death, including ferroptosis. Indeed, the heat shock protein family B member (HSPB1, HSP27) and the heat shock 70 kDa protein 5 (HSPA5, GRP78, BIP) were reported to be negative regulators of ferroptosis in cancer cells that were mediated by various mechanisms, namely, decreased iron uptake and GPX4 stabilization, respectively.^70,71^ One can also suggest that MNP@CD26@17D-induced ferroptosis may be due to the potentiated inhibitory effect on HSP90 expression compared to the HSP90 inhibitory action of the free drug 17-DMAG (Figure 3f). However, the role of different classes of HSP90 inhibitors during ferroptotic cell death may be more complex. For example, it has been shown that 2-amino-5-chloro-N,3-dimethylbenzamide (CDDO), an HSP90 inhibitor, can limit necroptosis and ferroptosis, two necrotic cell death mechanisms by inhibiting RIP1 kinase activity and blocking GPX4 degradation, respectively, in cancer cells.^72^

**Figure 7:**
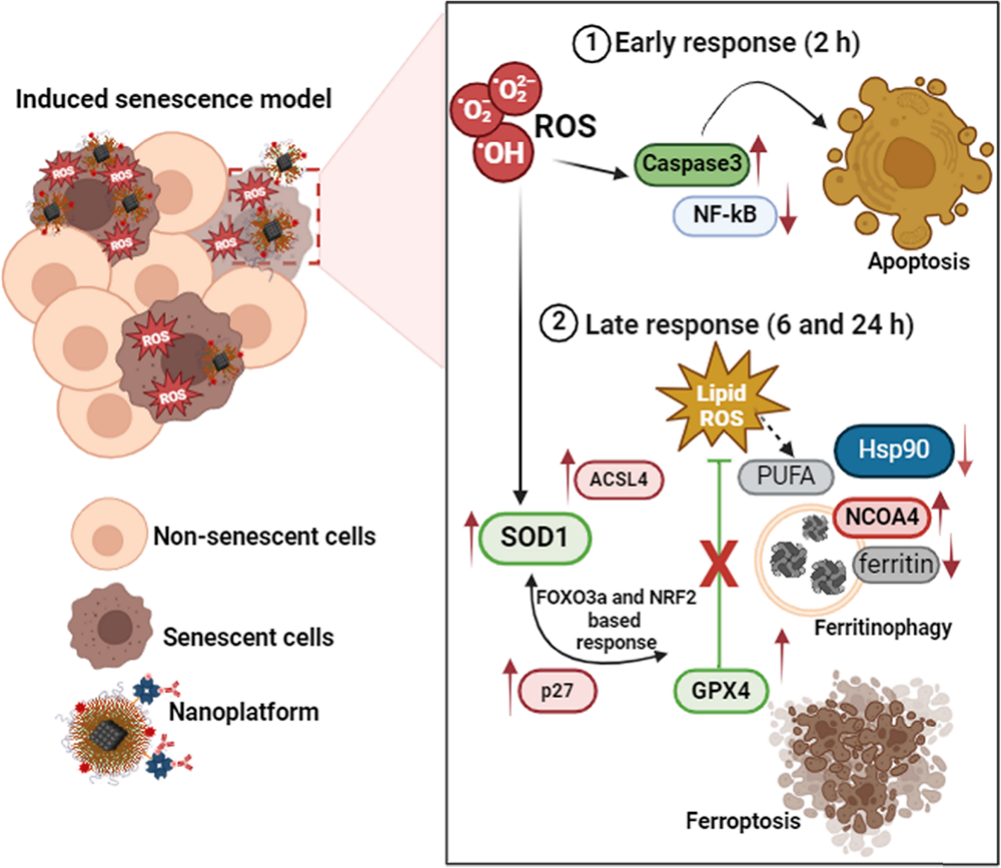
Dual senolytic response to MNP@CD26@17D treatment in senescent skin A431 cancer cells. Senescence was induced by etoposide treatment. Short-term incubation with MNP@CD26@17D promoted apoptotic cell death, whereas long-term treatment stimulated lipid peroxidation-mediated ferroptotic cell death in senescent skin cancer cells.

In conclusion, we have shown that CD26 levels can be elevated in drug-induced senescent skin cancer cells and that it can be targetable using anti-CD26 antibodies attached to a novel nanobased drug delivery system. We have shown that targeted delivery of an HSP90 inhibitor senolytic drug using the multifunctional nanoplatform may overcome apoptosis resistance and promote cell death in senescent skin cancer cells (Figure 7), with an efficiency higher than that of the drug alone.

Initially, the apoptosis-based elimination of senescent skin cancer cells was observed. Prolonged treatment with multifunctionalized nanoplatform also promoted lipid peroxidation-mediated cell death ferroptosis (Figure 7).

We postulate that the nanoplatform-associated ferroptotic cell death may be stimulated by the induction of a form of selective autophagy, here, ferritinophagy, as judged by elevated levels of NCOA4, a ferritinophagic marker, and a decreased pool of ferritin, an iron storage protein. Hemocompatibility of MNP@CD26@17D was also initially documented using the erythrocyte model *in vitro*, suggesting that MNP@CD26@17D treatment is generally safe when used up to 24 h. However, more studies are needed to establish the universal senolytic potential of MNP@CD26@17D in different types of senescent cancer cells with elevated levels of the cell surface marker CD26.

## Data Availability

The data presented in this study are available in the Supporting Information.

## Supporting Information Available

(Figure S1) Analysis of gene mutations in the functional group of surface receptors in A431 cells; (Figure S2) hysteresis curve for MNP@PMAO at 5 and 300 K; (Table S1) magnetic properties of MNP@PMAO nanoparticles; (Figure S3) TGA curves of the MNP@OA and MNP@PMAO; (Figure S4) FTIR spectra of MNP@OA and MNP@PMAO samples; (Section S1) theoretical estimation of the amount of streptavidin-conjugated on the MNP surface; (Figure S5) oxygen uptake curves of methyl linoleate emulsion with addition of MNPs/17D at 37 °C; (Table S2) rate of oxidation of LinMe (2.74 mM) in Triton-X micelles (8 mM) in PBS pH = 7.4 containing 10 mM AAPH (ABAP) with and without (blank) addition of MNPs/17D at 37 °C; (Figure S6) analysis of hemocompatibility of MNP@CD26@17D; (Figure S7) analysis of apoptosis parameters upon 17-DMAG, MNP@CD26, and MNP@CD26@17D treatment for 24 h in oxidant-induced senescent WI-38 and BJ fibroblasts; analysis of FOXO3a and ACSL4 levels in oxidant-induced senescent BJ cells upon 17-DMAG, MNP@CD26, and MNP@CD26@17D treatment for 2 and 6 h; analysis of transferrin receptor (CD71, hTfR1, TfR) levels in drug-induced senescent skin cancer A431 cells and the correlation between the levels of CD71 and senescence-associated beta-galactosidase activity; (Figure S8) analysis of gene mutations within selected functional gene groups relevant to responses to anticancer drugs such as apoptosis, autophagy, and oxidative stress; analysis of gene mutation types within apoptosis functional group of genes; (Figure S9) analysis of gene mutation types within oxidative stress functional group of genes; (Figure S10) analysis of gene mutation types within the iron metabolism functional group of genes and list of the mutated gene set in A431 skin cancer cell line (genes related to autophagy, ferroptosis, necroptosis, apoptosis, oxidative stress, cancer, surface receptors, and iron metabolism) (ZIP)
