# Supplementary material for: Design of a Magnetic Nanoplatform Based on CD26 Targeting and HSP90 Inhibition for Apoptosis and Ferroptosis-Mediated Elimination of Senescent Cells
Source: ACS Biomater Sci Eng. 2024 Dec 4;11(1):280–97. doi: 10.1021/acsbiomaterials.4c00771 (PMC11733919; doi:10.1021/acsbiomaterials.4c00771)
Supplement: Supplementary file 1 — ab4c00771_si_001.zip [file ab4c00771_si_001.zip › Supporting Information/Supporting Information.docx]

**Supporting Information**

**Design of a magnetic nanoplatform based on CD26 targeting and HSP90 inhibition for apoptosis and ferroptosis-mediated elimination of senescent cells**

Maciej Wnuk^a^#, Susel Del Sol-Fernández^b^#, Dominika Błoniarz^a^, Julia Słaby^c^, Tomasz Szmatoła^d^, Michał Żebrowski^e^, Pablo Martínez-Vicente^b^, Grzegorz Litwinienko^e^, María Moros^b,f^*, Anna Lewińska^a^*

*^a^Institute of Biotechnology, College of Natural Sciences, University of Rzeszow, Pigonia 1, 35-310 Rzeszow, Poland*

*^b^Instituto de Nanociencia y Materiales de Aragón, INMA (CSIC-Universidad de Zaragoza), C/ Pedro Cerbuna 12, 50009, Zaragoza, Spain*

*^c^Doctoral School, University of Rzeszow, Rejtana 16C, 35-959 Rzeszow, Poland*

*^d^Center of Experimental and Innovative Medicine, University of Agriculture in Krakow, al. Mickiewicza 24/28, 30-059 Cracow, Poland*

*^e^Faculty of Chemistry, University of Warsaw, Pasteura 1, 02-093 Warsaw, Poland*

*^f^Centro de Investigación Biomédica en Red de Bioingeniería, Biomateriales y Nanomedicina (CIBER-BBN), 28029, Madrid, Spain*

#First co-authorship

*Senior co-authorship

*Correspondence: María Moros ([mamoros@unizar.es](mailto:mamoros@unizar.es)), Anna Lewińska ([alewinska@ur.edu.pl](mailto:alewinska@ur.edu.pl)).

**
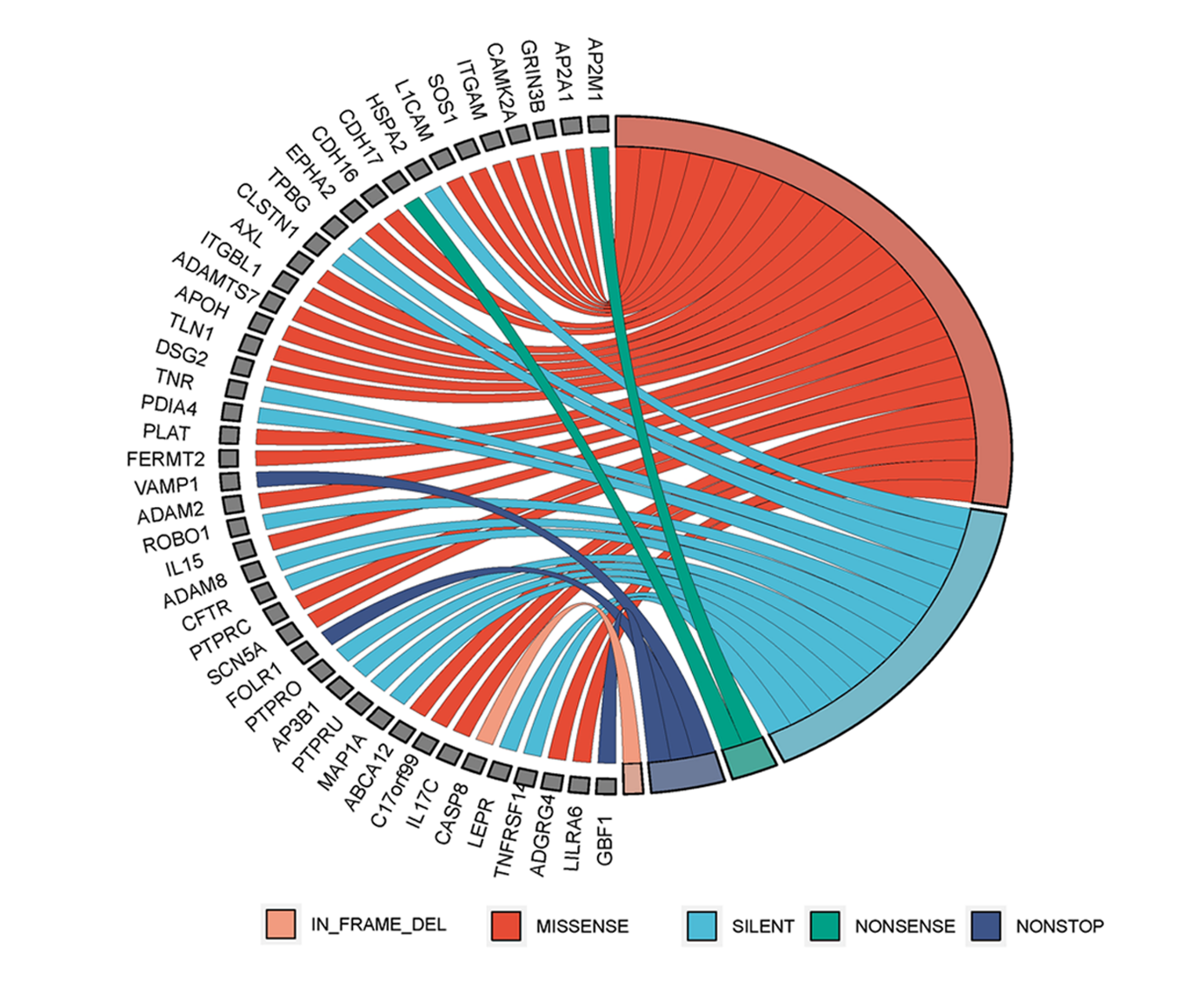
**

**Figure S1.** Analysis of gene mutations in the functional group of surface receptors in A431 cells. Gene mutations in *DPP4* gene were not identified. Gene mutation raw data were acquired from DepMap portal (<https://depmap.org/portal/>). Gene mutation types in the functional group of surface receptors are presented as a chord diagram (<https://www.bioinformatics.com.cn>).





**Figure S2.** Hysteresis curve for MNP@PMAO at 5 and 300 K. The magnetic response was recorded as a function of the applied field at 5 and 300 K.

The ferrimagnetic behaviour of the sample at low temperature is shown in **Table S1**. However, as temperature rises both the coercive field and the remanent magnetization drop to near zero, indicating the typical superparamagnetic behaviour of iron oxide nanoparticles.

**Table S1.** Magnetic properties of MNP@PMAO nanoparticles.

| **Temperature (K)** | ***M*_S_^a^**  (emu/g) | ***M*_r_**  (emu/g) | ***H_C_***  (Oe) |
| --- | --- | --- | --- |
| **300** | 77.0 | 8.5 | 9.0 |
| **5** | 80.0 | 16.4 | 305.0 |

^a)^ Determined with organic content correction based in TGA curves.





**Figure S3.** TGA curves of the MNP@OA and MNP@PMAO. The organic content is considerably increased after polymer addition, in accordance with the higher molecular weight of PMAO and the multilayer adsorption of OA chains over the inorganic surface.


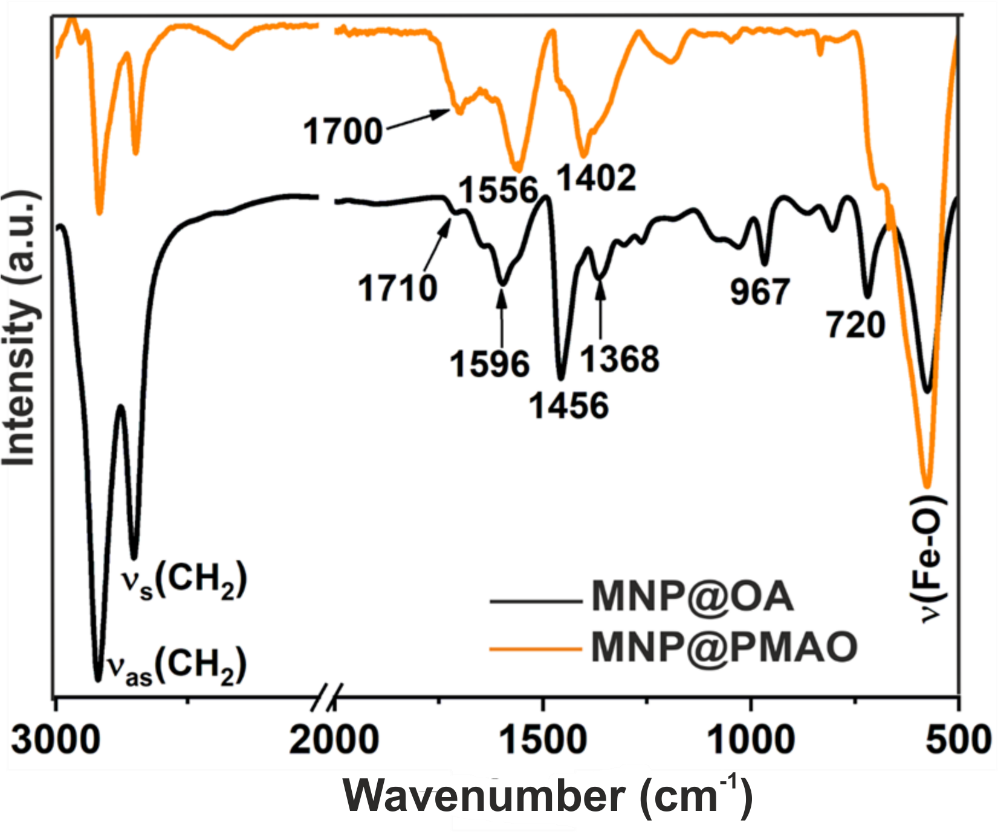


**Figure S4**. FTIR spectra of MNP@OA and MNP@PMAO samples.

For MNP@OA sample, the profile is dominated by methylene signals of oleic acid chains with bands at 2923 and 2852 cm^-1^ for ν_a_(CH_2_) and ν_s_(CH_2_) modes, respectively. The peak at 1596 cm^-1^ accounts for the ν_a_(CO_2_^-^) mode, while the symmetric one is rather difficult to locate because it is masked by the contribution of the scissoring δ(CH_2_) mode at 1465 cm^-1^. However, we assume that the shoulder around 1422 cm^-1^ corresponds to this mode, which makes splitting (Δ) of 174 cm^-1^. Hence, the carboxylate groups in the iron oxide nanoparticles are likely coordinated to iron ions forming a bridging bidentate complex. The C=O stretching around 1700-1710 cm^-1^ occurs when the oleic acid is forming dimers. Also, a signal centred at 587 cm^-1^ appears, which is characteristic of the Fe-O lattice vibrations in magnetite, indicating the formation of this iron oxide phase. As we can see, the Fe-O vibration was retained after polymer addition (MNP@PMAO). It is worth noting the appearance of a medium intensity band between 1700-1710 cm^-1^ (with a small shoulder around 1760 cm^-1^) in the region of the carbonyl C=O stretching. This is due to the presence of a secondary layer of oleic acid physically adsorbed on the primary one by interactions between the nonpolar tails.

**Section 1.** **Theoretical estimation of the amount of streptavidin-conjugated on the MNP surface**

To estimate the amount of protein molecules per nanoparticle, we consider the particle as a cube to simplify the calculations, so its volume is given by:

$$Vp=a^{3}$$

where, the diameter from TEM is D = 12.1 nm, so the volume of each nanoparticle is:

$Vp=$ 1.77 x ${10}^{-18}$ cm^-3^

Then, considering the density of Fe_3_O_4_ as δ = 4.9 g/cm^3^, the mass of 1 MNP is:

$$m_{MNP}=8.68 x {10}^{-18}g$$

From this, we can easily arrive to $m_{total}=0.63 mg$ and the number of MNP as:

$$N_{MNP}=\frac{m_{total}}{m_{MNP}}=7.26 x {10}^{13}$$

From Bradford assay we quantified the amount of STV in the supernatant after MNP@STV, which is:

SN1= 1.5 mg/ml and SN control= 36.8 mg/ml.

The MW of STV = 52.8 kDa, so $N_{STV}=4.0 x {10}^{14}$

Finally, the amount of STV per MNP is: ~ 6 STV/MNP.

**
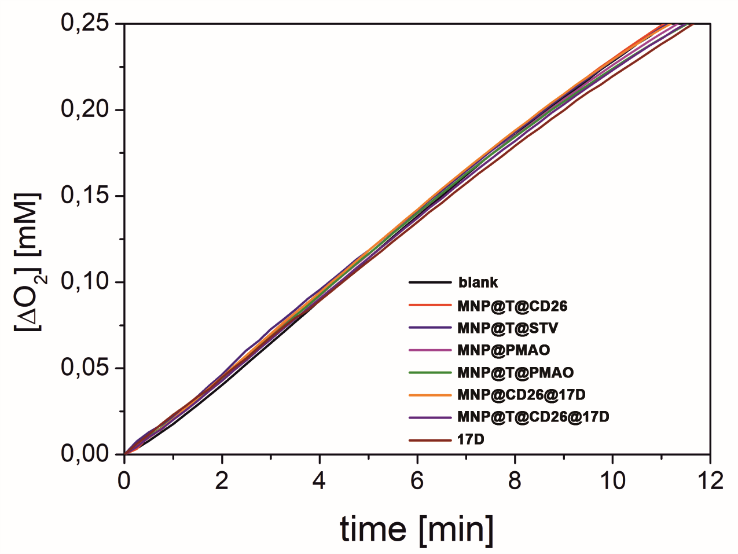
**

**Figure S5.** Oxygen uptake curves of methyl linoleate emulsion with addition of MNPs/17D at 37°C. Samples were abbreviated according to Scheme 1 (main text).

**Table S2.** Rate of oxidation of LinMe (2.74 mM) in Triton-X micelles (8 mM) in PBS pH = 7.4 containing 10 mM AAPH (ABAP) with and without (blank) addition of MNPs/17D at 37°C. Samples were abbreviated according to Scheme 1 (main text).

| No. | Sample | C | | *R*_ox1_ [nM s^-1^] |
| --- | --- | --- | --- | --- |
| 0 | blank | | - | 350 ± 28 |
| 1 | MNP@T@CD26 | | 3 µg/ml | 354 ± 23 |
| 2 | MNP@T@STV | | 3 µg/ml | 368 ± 5 |
| 3 | MNP@PMAO | | 3 µg/ml | 379 ± 24 |
| 4 | MNP@T@PMAO | | 3 µg/ml | 378 ± 4 |
| 5 | MNP@CD26@17D | | 3 µg/ml | 380 ± 5 |
| 6 | MNP@T@CD26@17D | | 3 µg/ml | 363 ± 18 |
| 7 | 17D | | 1 µM | 348 ± 8 |

**

**

**Figure S6.** Analysis of hemocompatibility of MNP@CD26@17D. Hemocompatibility test was assayed using an erythrocyte model. Potassium chloride treatment served as a positive control, 100% hemolysis (PC). NC, non-treated erythrocytes. Bars indicate SD, n = 3, ^***^*p* < 0.001, ^**^*p* < 0.01 compared to PC (ANOVA and Dunnett’s a posteriori test), ^###^*p* < 0.001, ^##^*p* < 0.01 compared to NC (ANOVA and Dunnett’s a posteriori test).

**
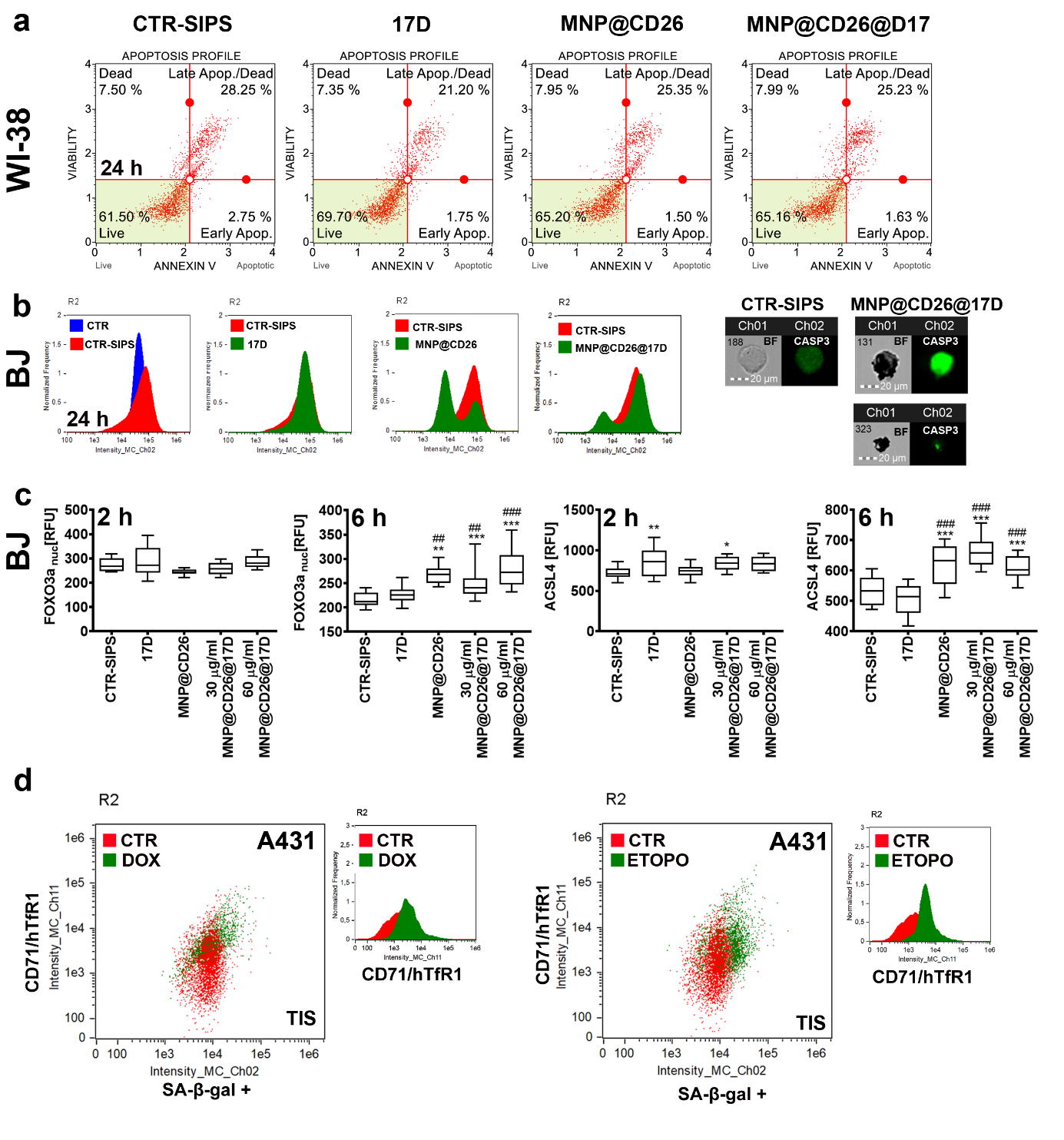
**

**Figure S7**. (a, b) Analysis of apoptosis parameters upon 17-DMAG, MNP@CD26, and MNP@CD26@17D treatment for 24 h in oxidant-induced senescent WI-38 (a) and BJ (b) fibroblasts. (a) Phosphatidylserine externalization was assayed using flow cytometry and Annexin V staining. (b) Caspase 3 levels were analyzed using imaging flow cytometry using a dedicated anti-caspase 3 antibody (Ch02, green signals). Representative dot-plots and histograms are presented, respectively. (c) Analysis of FOXO3a and ACSL4 levels in oxidant-induced senescent BJ cells upon 17-DMAG, MNP@CD26, and MNP@CD26@17D treatment for 2 and 6 h. FOXO3a and ACSL levels were assayed using imaging cytometry and dedicated antibodies. The levels of analyzed proteins are presented as relative fluorescence units (RFU). Box and whisker plots are shown, n = 3, ^***^*p* < 0.001, ^**^*p* < 0.01, ^*^*p* < 0.05 compared to senescence control (CTR-SIPS, ANOVA and Dunnett’s a posteriori test), ^###^*p* < 0.001, ^##^*p* < 0.01, compared to 17-DMAG alone (17D). SIPS, stress-induce premature senescence; 17D, 17-DMAG treatment; MNP@CD26, nanoplatform containing anti-CD26 antibody; MNP@CD26@17D, nanoplatform containing anti-CD26 antibody and 17-DMAG. (d) The analysis of transferrin receptor (CD71, hTfR1, TfR) levels in drug-induced senescent skin cancer A431 cells (DOX, doxorubicin treatment; ETOPO, etoposide treatment) and the correlation between the levels of CD71 and senescence-associated beta-galactosidase activity (SA-β-gal+). Imaging cytometry, a dedicated anti-CD71 antibody and SA-β-gal kit were used. Representative dot-plots and histograms are presented.


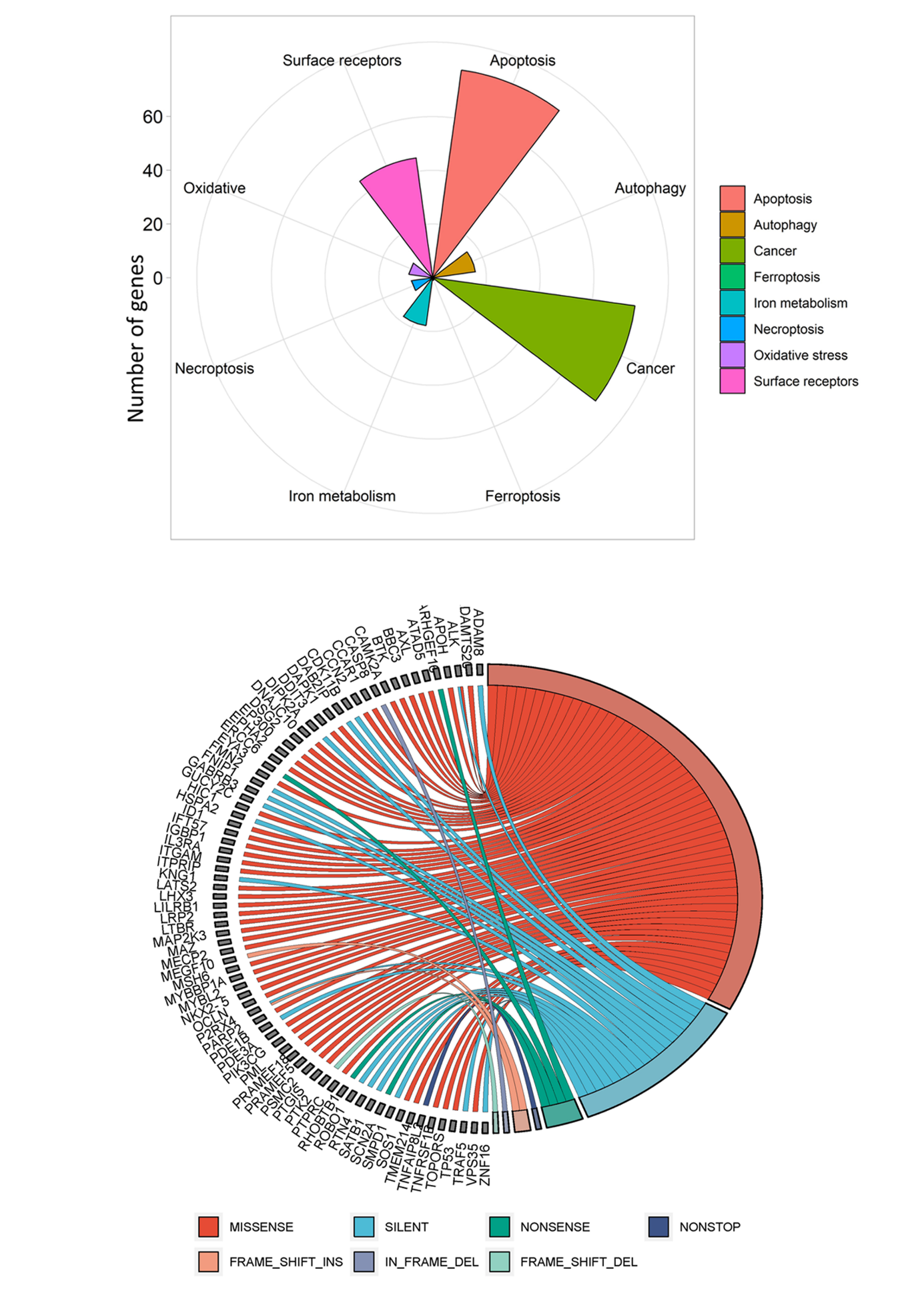


**Figure S8**. (Top) Analysis of gene mutations within selected functional gene groups relevant to responses to anticancer drugs such as apoptosis, autophagy, oxidative stress, etc. (Bottom) Analysis of gene mutation types within apoptosis functional group of genes. Gene mutation raw data were acquired from DepMap portal (<https://depmap.org/portal/>). (Top) The number of mutated genes is presented as a radial bar chart. (Bottom) Gene mutation types are presented as a chord diagram (<https://www.bioinformatics.com.cn>).


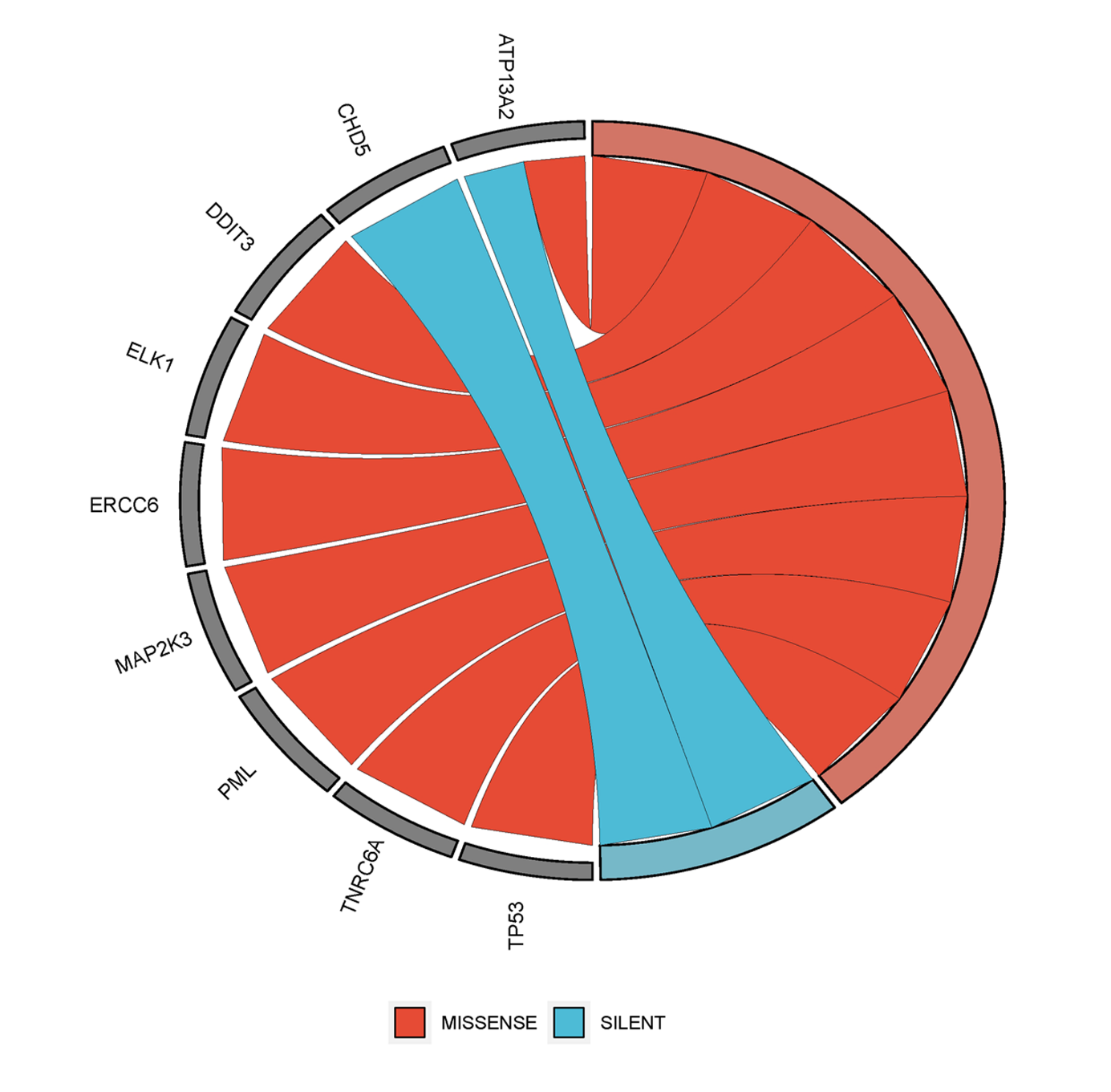


**Figure S9.** Analysis of gene mutation types within oxidative stress functional group of genes. Gene mutation raw data were acquired from DepMap portal (<https://depmap.org/portal/>). Gene mutation types are presented as a chord diagram (<https://www.bioinformatics.com.cn>).


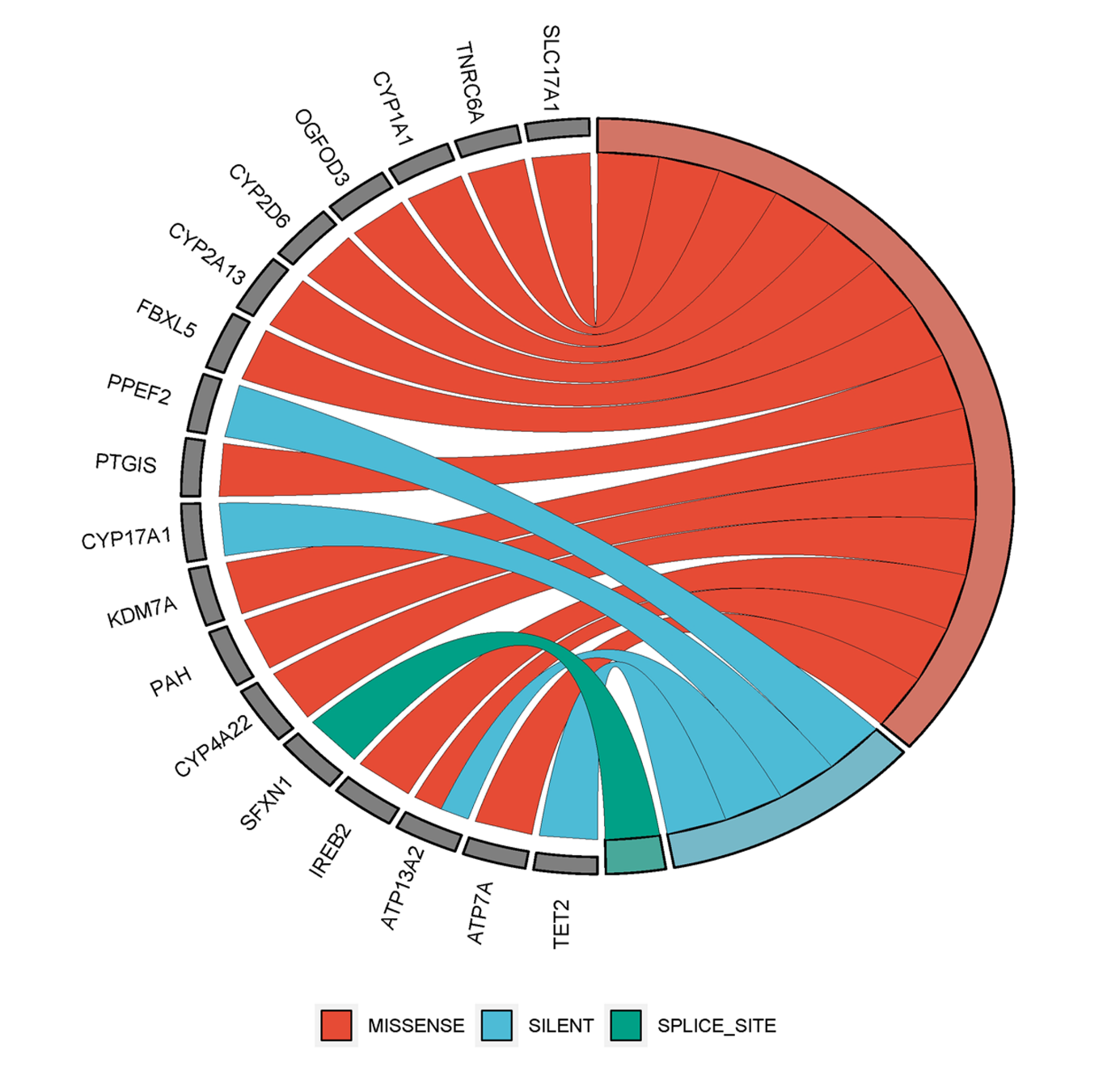


**Figure S10.** Analysis of gene mutation types within iron metabolism functional group of genes. Gene mutation raw data were acquired from DepMap portal (<https://depmap.org/portal/>). Gene mutation types are presented as a chord diagram (<https://www.bioinformatics.com.cn>).
